# Reaction between Indazole and Pd-Bound Isocyanides—A Theoretical Mechanistic Study

**DOI:** 10.3390/molecules23112942

**Published:** 2018-11-10

**Authors:** Girolamo Casella, Maurizio Casarin, Vadim Yu. Kukushkin, Maxim L. Kuznetsov

**Affiliations:** 1 Dipartimento di Scienze della Terra e del Mare, Università degli Studi di Palermo, Via Archirafi, 22, 90123 Palermo, Italy; girolamo.casella@unipa.it; 2 Consorzio Interuniversitario di Ricerca in Chimica dei Metalli nei Sistemi Biologici (C.I.R.C.M.S.B.), Piazza Umberto I, 70121 Bari, Italy; 3 Dipartimento di Scienze Chimiche, Università di Padova, via F. Marzolo 1, 35131 Padova, Italy; maurizio.casarin@unipd.it; 4 International Group on Organometallic Chemistry, Institute of Chemistry, Saint Petersburg State University, Universitetskaya Nab., 7/9, Saint Petersburg 199034, Russia; v.kukushkin@spbu.ru; 5 Centro de Química Estrutural, Instituto Superior Técnico, Universidade de Lisboa, Av. Rovisco Pais, 1049-001 Lisbon, Portugal

**Keywords:** isocyanides, nitriles, nucleophilic addition, DFT calculations, activation of small molecules, reaction mechanism

## Abstract

The mechanism of the addition of indazole (Ind)—a bifunctional aromatic N,NH-nucleophile—to cyclohexyl isocyanide coordinated to the palladium(II) center in the model complex *cis*-[PdCl_2_(CNMe)(CNCy)] (**1**) to give the corresponding aminocarbene ligand was investigated in detail by theoretical (DFT) methods. The most plausible mechanism of this reaction is that of the associative type involving nucleophilic attack of Ind by its unprotonated N atom at the isocyanide carbon atom followed by the stepwise proton transfer from the nucleophile molecule to the isocyanide N atom via deprotonation/protonation steps. Two reaction channels based on two tautomeric forms of indazole were found. The channel leading to the experimentally isolated aminocarbene product is based on the less stable tautomeric form. Another channel based on the more stable tautomer of Ind is slightly kinetically more favorable but it is endergonic. Thus, the regioselectivity of this reaction is thermodynamically rather than kinetically driven. The bonding situation in key species was analyzed.

## 1. Introduction

The coupling of *N*-nucleophiles and isocyanides (C≡NR) is an important type of organic transformation yielding a great variety of chemical systems with newly formed C–N bonds [1,2,3,4]. In particular, these reactions represent an excellent route toward acyclic diaminocarbenes—an attractive alternative to *N*-heterocyclic carbene- and phosphine-based metal complexes used as catalysts in a number of important chemical processes [5,6,7,8,9,10,11], for example, Heck [12,13], Suzuki [12,14], Suzuki-Miyaura [13,15,16,17], and Sonogashira [13,18,19] cross-coupling reactions and some cyclizations/additions to substrates featuring C≡C and C=C bonds [20,21,22,23,24].

Due to the high chemical inertness of isocyanides toward nucleophilic addition (NA), they should usually be activated, e.g., by coordination to an appropriate metal center. Addition of NH-nucleophiles with the sp^3^-type nitrogen orbital hybridization (i.e., mono- or bifunctional amines [20,21,22,25,26,27,28,29,30,31,32,33,34,35,36,37,38,39,40,41,42,43,44,45,46,47,48,49,50,51] and hydrazines [17,52,53,54,55,56,57,58,59]) to metal-bound isocyanides was extensively reported in the literature. Examples of NA of sp^2^-*N*-nucleophiles (e.g., imines [60]) and mixed sp^2^/sp^3^-*N*-nucleophiles (e.g., hydrazones [15] and amidines [18,61]) are much rarer.

**Scheme 1:**
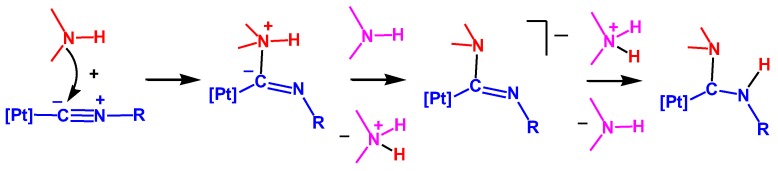
The mechanism of nucleophilic addition (NA) of amines, imines, and hydrazones to isocyanides coordinated to the Pt(II) center.

Despite intensive experimental investigations of NA of *N*-nucleophiles to metal coordinated isocyanides, the mechanism of this addition started to become clear only recently. The experimental kinetic studies [43,62,63] indicated a negative activation entropy for the reaction between amines and isocyanides ligated to the Pt(II) or Pd(II) centers. However, as recently as 2017, it was established by theoretical calculations that the mechanism of NA of amines, imines, and hydrazones (HNMe_2_, HN=CPh_2_, and H_2_N–N=CPh_2_) to the platinum(II) complexes *cis*-[Pt(C≡NCy)(2-pyz)(dppe)]^+^ (2-pyz = 2-pyrazyl, dmpe = Me_2_PCH_2_CH_2_PMe_2_) and *cis*-[PtCl_2_(C≡NXyl)(C≡NMe)] is of the stepwise associative type including an attack of the nucleophile by the NH nitrogen atom at the isocyanide carbon atom followed by the amino or imino proton migration via stepwise deprotonation/protonation steps, as shown in Scheme 1 [64].

**Scheme 2:**
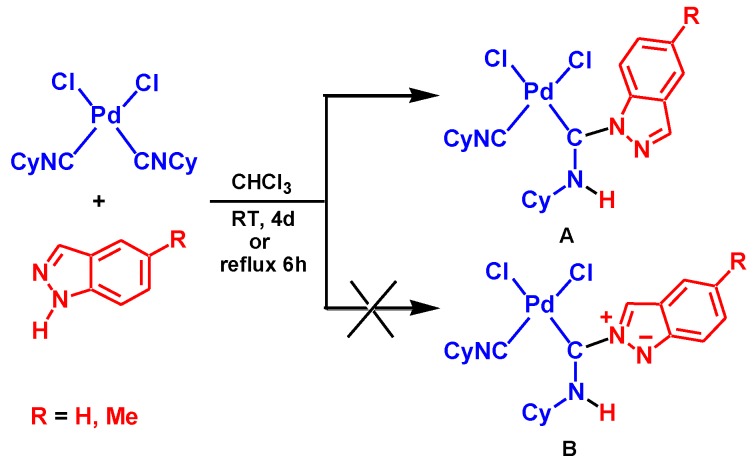
Reaction between *cis*-[PdCl_2_(C≡NCy)_2_] and the indazoles.

Coupling between aromatic heterocycles with the pyrazole unit and metal-activated isocyanides was reported only recently [65]. The reaction between equimolar amounts of indazole (Ind) or 5-methylindazole and the complex *cis*-[PdCl_2_(CNCy)_2_] (Cy = cyclohexyl) in chloroform yields aminocarbene species **A**, see Scheme 2. However, details of the mechanism for this reaction were not studied. In particular, the nature of the reaction regioselectivity is unclear. The Indazole molecule has two adjacent imine and amine nucleophilic centers. The former center exhibits stronger nucleophilic properties compared to the latter one. Indeed, in accord with DFT calculations (see Computational Details section), the cationic indazole molecule protonated at the imino N atom is more stable than Ind protonated at the amino nitrogen by 20.8 kcal/mol. Therefore, the preferable formation of a product of the **B** type, see Scheme 2, would be expected as a result of this reaction but only product **A** was isolated experimentally from the reaction mixture.

With the aim of interpreting these curious experimental findings and investigating in detail the mechanism of the reaction, theoretical calculations at the DFT (M06L) level of theory have been undertaken for the coupling of indazole and cyclohexyl isocyanide ligand in the model complex *cis*-[PdCl_2_(CNMe)(CNCy)] (**1**), and results of this study are reported here.

## 2. Computational Details

### 2.1. Calculations of the Reaction Mechanism

The full geometry optimization of all structures and transition states (TSs) was carried out at the DFT level of theory by using the M06L functional [66] with the help of the Gaussian 09 [67] program package. No symmetry operations were applied. The geometry optimization was carried out by using a quasi-relativistic Stuttgart pseudopotential that describes 28 core electrons and the appropriate contracted basis set (8s7p6d)/[6s5p3d] [68] for the palladium atom and the 6-31G* basis set for other atoms. Single-point calculations were then performed on the basis of the equilibrium geometries found by using the 6-311+G** basis set for non-metal atoms. The solvent effects were taken into account in both optimization and single-point calculations using the Solvation Model based on Density (SMD) [69] with chloroform taken as solvents. The energies discussed below are Gibbs free energies G(6-311+G**) calculated as G(6-311+G**) = E(6-311+G**) – E(6-31G*) + G(6-31G*) where the basis set used is indicated. This combination of method and basis sets was tested in our previous work for NA of amines, imines, and hydrazones to metal-bound isocyanides [64] and provided excellent agreement with available experimental data [62], the DFT calculated and experimental activation enthalpies being 6.9 and 7.0 ± 0.8 kcal/mol, respectively, and the corresponding Gibbs free energies of activation being 21.6 and 19.8 ± 1.7 kcal/mol for the reaction between *cis*-[Pt(C≡NCy)(2-pyz)(dppe)]^+^ and HNEt_2_.

The Hessian matrix was calculated analytically for the optimized structures to prove the location of correct minima (no imaginary frequencies) or saddle points (only one imaginary frequency) and to estimate the thermodynamic parameters, with the latter calculated at 25 °C. Transition states were calculated using a Berny geometry optimization and force constants calculated analytically for the first points. The starting geometries for the TS optimization were found with the help of the QST3 algorithm and/or potential energy scans. The nature of all transition states was investigated by analysis of the vectors associated with the imaginary frequency and by the calculations of the intrinsic reaction coordinates (IRC) by using the method developed by Gonzalez and Schlegel [70,71,72].

### 2.2. Bond Analysis

Single-point calculations on the previously optimized geometries of compounds **1**, **TS6**, **TS14**, *Z*-**P2**, and *Z*-**P1** were carried out by using the ADF package [73,74] in the DFT framework, at relativistic scalar ZORA level [75,76,77,78], by using the GGA BP86 functional [79,80,81] and Grimme3 BJ-DAMP dispersion corrections [82,83,84] in conjunction with the Slater-type triple ζ doubly polarized (TZ2P) basis set for all the atoms. Previously, it was shown that the GGA BP86 functional demonstrates very good performance in the analysis of the bonding nature in transition metal complexes [85], in the Voronoi Deformation Density (VDD) analysis [86], and in the Energy Decomposition Analysis (EDA) [87]. The effect of the solvent was taken into account according to the Conductor-like Screening Model (COSMO) formalism [88,89,90,91]. The bond analysis was carried out employing canonical Kohn-Sham molecular orbitals (KS-MO).

## 3. Results and Discussion

### 3.1. Reaction Mechanisms

**Scheme 3:**
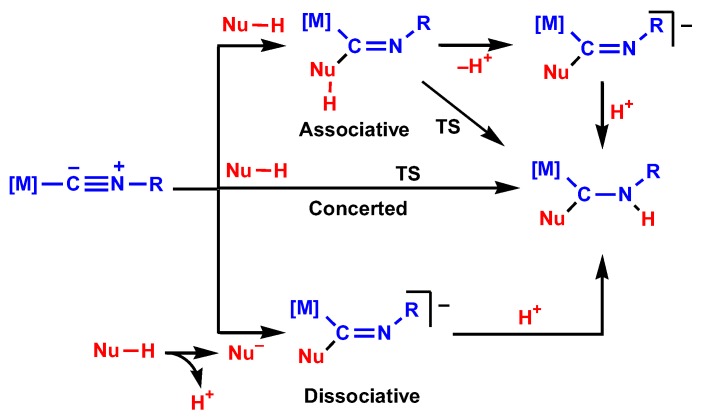
Mechanisms of nucleophilic addition to metal-bound isocyanides.

In this work, we consider three global types of mechanisms for NA to isocyanides, i.e., dissociative, concerted, and associative, see Scheme 3. The dissociative mechanism starts with deprotonation of the nucleophile by a base that is present in the reaction mixture. The deprotonated nucleophile attacks the isocyanide C atom to give an anionic intermediate, which is protonated to give the final reaction product. The concerted mechanism occurs in one step via a cyclic transition state, which may be either 4- or 6-membered with the participation of a third molecule (solvent, one of the reactants, or an additive) playing the role of a proton shuttle. The associative mechanism includes the addition of a nucleophile in the molecular form to the isocyanide C atom followed by proton transfer (concerted or stepwise) to the N atom. In the following section, all these mechanisms are discussed for the reaction between indazole and complex **1**.

### 3.2. Dissociative Mechanism

The strongest base in the reaction mixture is the imine N atom of indazole. Therefore, namely, this atom was considered as a base for the proton abstraction from indazole within the dissociative mechanism, see Reaction 1. The implicit solvation models (such as SMD used in this work) often fail to correctly describe solvent effects for the processes in which the number of species with the same charge is not preserved in the course of the reaction (as in the case of proton dissociation). Therefore, in order to estimate the energy of autodissociation of indazole (Reaction 1), the experimental pK_a_ values of indazole and its protonated form, IndH^+^, in water solution were used (13.86 and 1.25, respectively [92]). The ΔG values of the Ind and IndH^+^ dissociation (ΔG_2_ and ΔG_3_, reactions (2) and (3)) may be determined using equation ΔG = 2.303RTpK_a_, and they are 18.9 and 1.7 kcal/mol for ΔG_2_ and ΔG_3_, respectively. Then, the ΔG of autodissociation of indazole in water is ΔG_ad_ = ΔG_2_ – ΔG_3_ = 17.2 kcal/mol. The theoretically calculated value of ΔG_ad_ for reaction 1 in water is 28.1 kcal/mol, and the difference between theoretical and experimental values is 10.9 kcal/mol. The DFT calculated ΔG_ad_ value for the chloroform solution is 49.8 kcal/mol. Assuming that the deviation from the experimental data is the same for both water and chloroform solutions, the corrected ΔG_ad_ value for the latter is 39.0 kcal/mol. This energy is too high for the efficient occurrence of NA; therefore, the dissociative mechanism may be ruled out.


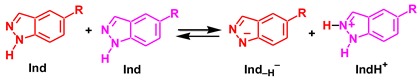
(1)  ΔG_ad_


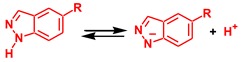
(2)              ΔG_2_


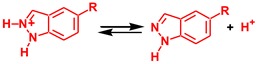
(3)             ΔG_3_

### 3.3. Concerted Mechanism

**Scheme 4:**
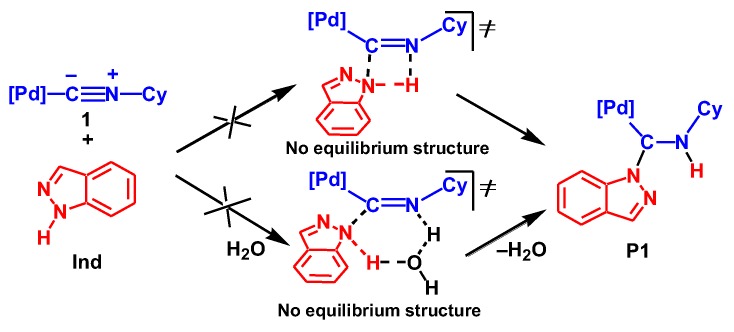
Concerted mechanism of the reaction between Ind and **1**.

This one-step mechanism should include the formation of a 4-or 6-membered cyclic transition state, see Scheme 4. However, careful search of the potential energy surface found no minimum corresponding to TS, which directly connects the reactants Ind + **1** and the final reaction product **P1**. All attempts of its location led to transition states of the associative mechanism discussed below.

### 3.4. Associative Mechanisms

Since the indazole molecule has two potential nucleophilic centers—the amino and imino nitrogen atoms—several associative-type pathways are possible.

#### 3.4.1. Nucleophilic Addition by the Amino Nitrogen Atom (Mechanism I)

**Scheme 5:**
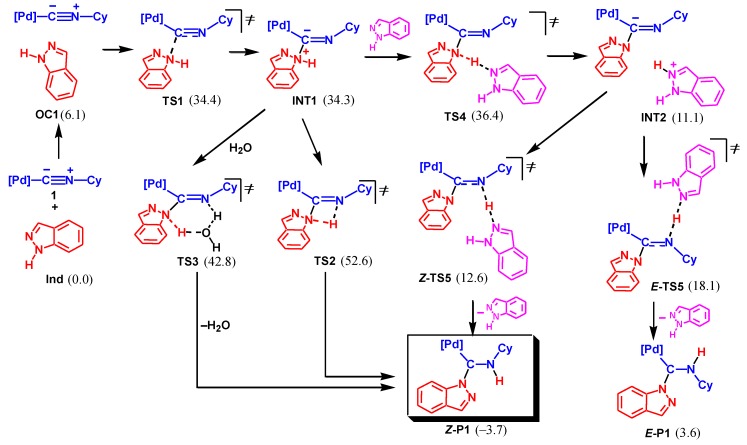
Associative mechanism of NA of indazole to **1** by amino nitrogen atom (Mechanism I, Gibbs free energies are indicated in kcal/mol relative to **1** + Ind, final product is boxed).

This mechanism is similar to that established previously for the reactions of amines, imines, and hydrazones to metal-bound isocyanides [64]. It starts with the formation of a van der Waals pre-reaction complex **OC1** which is then transformed into intermediate **INT1** via transition state **TS1** upon the attack of the amino N atom at the isocyanide C atom, see Scheme 5. The following proton transfer may occur either in a concerted- or a stepwise manner. Both 4- and 6-membered transition states were found for the concerted H-transfer (**TS2** and **TS3**). Water inevitably existing in the reaction mixture as moisture plays the role of a proton shuttle in **TS3**.

The more favorable stepwise proton transfer includes deprotonation of the indazole moiety in complex **INT1** by the second Ind molecule to give **INT2** via **TS4** and the subsequent protonation of the isocyanide N atom by IndH^+^ affording the final product **P1** through **TS5**. The protonation step may occur via two channels leading to either the *Z*- or *E*-isomer of the final product. The calculations indicate that the Z-channel is both kinetically and thermodynamically more favorable than the *E*-channel. These results are in agreement with the experimental isolation of the *Z*-isomer of **P1** from the reaction mixture [65].

As can be seen from Scheme 5, the proton abstraction step is the rate-limiting step for the whole process with the overall activation barrier of 36.4 kcal/mol (in terms of ΔG_s_^≠^). This value is too high to permit the realization of Mechanism I; therefore, the nucleophilic addition of Ind by the amino N atom may be ruled out. Thus, the mechanism of the reaction of indazole with the Pd-bound isocyanides is different from that when amines, imines, and hydrazones are employed as nucleophiles [64].

#### 3.4.2. Nucleophilic Addition by the Imino Nitrogen Atom (Mechanism II)

**Scheme 6:**
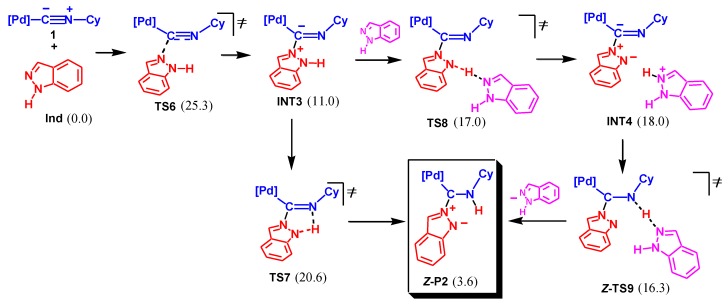
Associative mechanism of NA of indazole to **1** by imino nitrogen atom (Mechanism II, Gibbs free energies are indicated in kcal/mol relative to **1** + Ind, final product is boxed, only the *Z*-isomeric pathway is shown).

A similar associative mechanism was investigated for NA by the unprotonated imino N atom of Ind. The mechanism starts with the formation of intermediate **INT3** via **TS6**, as shown in Scheme 6. As in the previous case, the following proton shift may occur either in a concerted mode via the 5-membered transition state **TS7** or in a stepwise manner via **TS8**, intermediate **INT4**, and **TS9**. The latter pathway is slightly more energetically favorable than the former one (by 2.6 kcal/mol).

The rate-limiting step of this mechanism is the nucleophilic attack of Ind (via **TS6**), and the overall activation barrier is 25.3 kcal/mol. This value is quite reasonable and qualitatively corresponds to the experimental reaction conditions applied (20–25 °C for 4 days or under reflux in CHCl_3_ for 6 h [65]). However, the final product of this pathway is complex **P2**, which was not detected experimentally. In accord with the calculations, the formation of **P2** from Ind and **1** is endergonic by 3.6 kcal/mol. Applying the experimental conditions (initial concentrations c_0_[1] = c_0_[Ind] ≈ 20 mmol/l), such a ΔG_s_ value should correspond to the maximum concentration of **P2** of ca. 8 × 10^−4^ mol/l. Thus, to detect the formation of **P2** in a solution, a special instrumental technique, which was not applied in reference [65], should be used. Thus, despite favorable kinetics, the reaction Ind + **1** → **P2** is not feasible thermodynamically.

#### 3.4.3. Isomerization of **P2** into **P1**

**Scheme 7:**
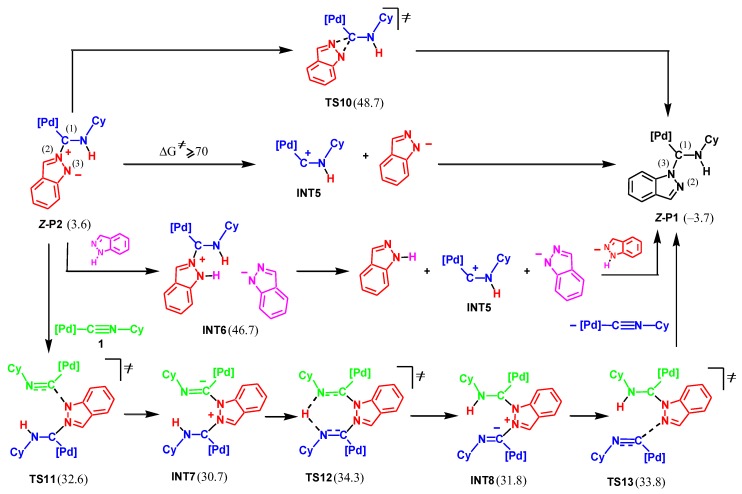
Possible mechanisms of isomerization of **P2** into **P1** (Gibbs free energies relative to **1** + Ind are shown in kcal/mol).

Since the final product of the reaction is **P1**, it may be formed as a result of the isomerization of **P2** into **P1**. Four possible pathways were considered for this transformation, as shown in Scheme 7.

**(i) Monomolecular isomerization.** Such isomerization occurs in one step via the formation of a three-membered cyclic transition state, **TS10**. However, the activation energy of this route is too high (45.1 kcal/mol).

**Figure 1:**
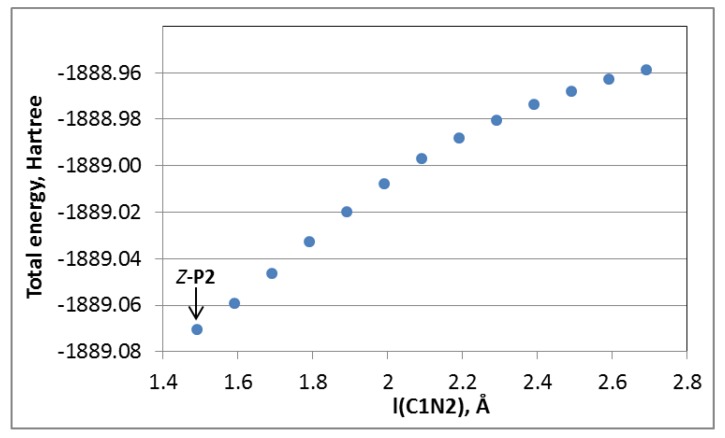
Energy scan for the C(1)N(2) bond cleavage in *Z*-**P2**.

**(ii) Stepwise rebound.** This pathway includes the cleavage of the C(1)N(2) bond in *Z*-**P2** to give the cationic complex, **INT5**, and the deprotonated indazole, Ind_-H_^–^, followed by the formation of the C(1)N(3) bond. We were unable to locate any transition state for the C(1)N(2) bond cleavage. However, results of the energy scan toward this process, see Figure 1, indicate that the lowest limit of the activation energy is 70 kcal/mol (in terms of activation enthalpy at 0 K). Even considering the favorable entropic factor of this step, the activation barrier is too high, and this pathway may be excluded.

**(iii) Proton-assisted stepwise rebound.** The C(1)N(2) bond cleavage may be facilitated by the protonation of the indazole moiety in **P2**. However, the protonation step is energetically unfavorable, with the ΔG_s_ value of formation of intermediate **INT6** being 43.1 kcal/mol.

**(iv) Stepwise rebound assisted by complex 1.** The C(1)N(2) bond rupture may also be assisted by the second molecule of complex **1**. This pathway includes the attack of **P2** by the N(3) atom at the isocyanide carbon atom of the second molecule of **1** to give **INT7** via **TS11**. The following proton transfer and C(1)N(2) bond cleavage leads to the final product **P1** (via **TS12**, **INT8**, and **TS13**). However, the high activation barrier (30.7 kcal/mol) disproves this mechanism. Thus, the formation of the final product **P1** through isomerization of **P2** may be ruled out.

#### 3.4.4. Mechanism Involving the Less Stable Tautomeric Form of Indazole (Mechanism III)

**Scheme 8:**
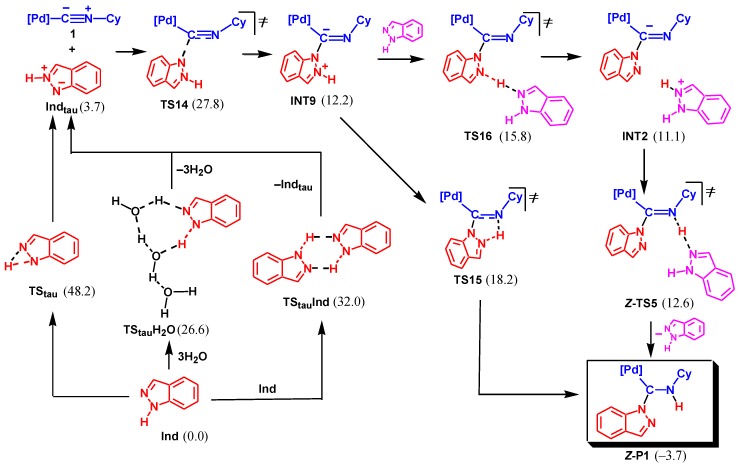
Associative mechanism of NA of indazole to **1** based on the less stable tautomeric form of Ind (Mechanism III, Gibbs free energies are indicated in kcal/mol relative to **1** + Ind, final product is boxed, only the *Z*-isomeric pathway is shown).

Another possible mechanism is associated with the initial tautomerization of indazole and nucleophilic attack of the less stable tautomeric form at the isocyanide C atom, see Scheme 8. The monomolecular tautomerization via **TS_tau_** is not favorable with the activation barrier of 48.2 kcal/mol. However, the bimolecular tautomerization involving two Ind molecules via **TS_tau_Ind** requires significantly lower activation energy (32.0 kcal/mol). Finally, the proton shift assisted by water via **TS_tau_H_2_O** is even more favorable (ΔG_s_^≠^ = 26.6 kcal/mol). The tautomeric form **Ind_tau_** is only slightly endergonic relative to the most stable structure of Ind (by 3.7 kcal/mol).

The nucleophilic attack of **Ind_tau_** at the isocyanide of **1** results in the formation of **INT9** via **TS14**. The following proton transfer may occur either in a concerted fashion through the five-membered cyclic transition state, **TS15**, or in a stepwise manner via protonation/deprotonation involving Ind as a proton shuttle (**INT9** → **TS16** → **INT2** → **TS5** → **P1**).

The rate-limiting step of the whole mechanism is NA of **Ind_tau_** (via **TS14**) with an overall activation barrier of 27.8 kcal/mol. This value is only slightly higher than that found for Mechanism II (25.3 kcal/mol) leading to **P2**. However, the formation of **P1** is exergonic (by –3.7 kcal/mol) while the formation of **P2** is endergonic (by 3.6 kcal/mol). The difference of 7.3 kcal/mol for the ΔG_s_ of the two reaction channels, **1** + Ind → **P1** and **1** + Ind → **P2**, corresponds to the ratio of the equilibrium concentrations of **P1** and **P2** ca. 2.3 × 10^5^. Such a huge ratio perfectly explains the lack of experimental detection of product **P2** in the reaction mixture despite a slight kinetic preference for its formation over **P1**.

#### 3.4.5. Mechanisms based on the coordinated indazole

**Scheme 9:**
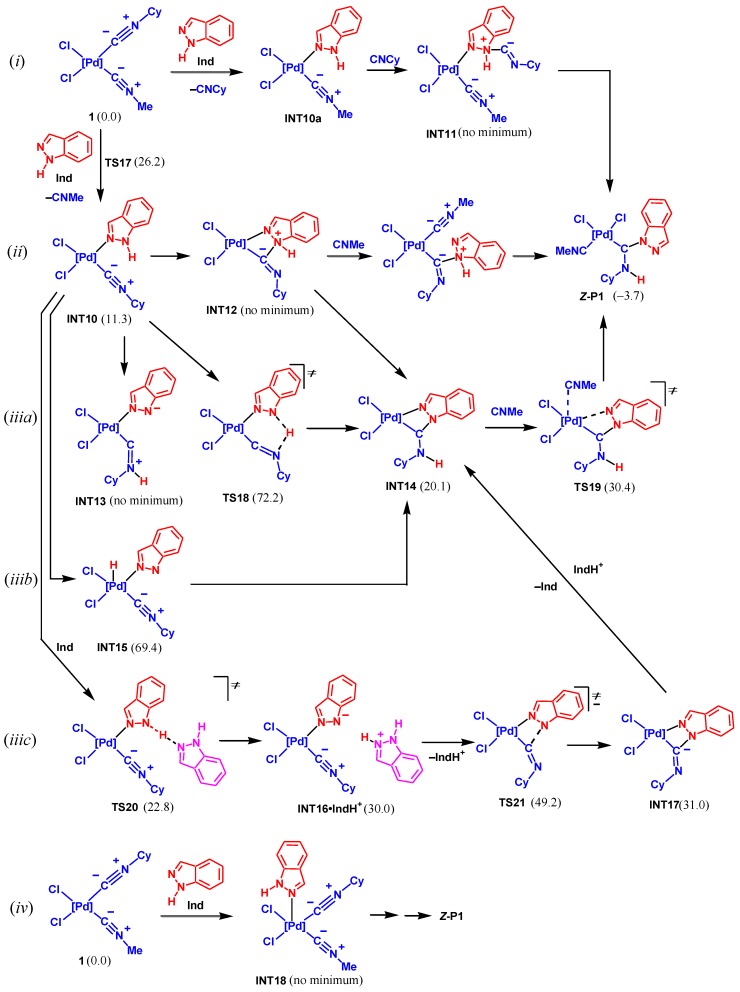
Mechanisms of NA of indazole to **1** based on the coordinated indazole (Gibbs free energies are indicated in kcal/mol relative to **1** + Ind).

This group of mechanisms includes the initial substitution of one of the isocyanide ligands for the indazole molecule to give complex **INT10**, as shown in Scheme 9. The substitution occurs via **TS17** in a concerted fashion with a rather high activation barrier of 26.2 kcal/mol, and it is endergonic by 11.3 kcal/mol. The further reaction may follow several possible pathways most of them being directly mediated by Pd, see Scheme 9.

*Pathway (i).* The coordinated indazole may attack the C atom of free isocyanide liberated upon the ligand substitution to give intermediate **INT11**. However, all attempts to locate the equilibrium structure of this intermediate failed to lead to **INT10a** and separate C≡NCy. Thus, this pathway is not feasible.

*Pathway (ii).* In this pathway, the coordinated indazole interacts with the coordinated isocyanide ligand to give the cyclic intermediate **INT12** followed by the Pd–N bond cleavage, coordination of the second isocyanide molecule, and proton transfer. However, the calculations indicated that there is no minimum corresponding to **INT12**, hence, this pathway may also be ruled out.

*Pathway (iiia).* In this and two following pathways, the deprotonation/proton transfer of/from the coordinated indazole precedes the C–N bond formation. The NH proton of indazole may be transferred to the N isocyanide atom via **TS18**. The calculations demonstrated that such a transfer is accompanied by the C–N bond formation to give **INT14**, while the acyclic intermediate **INT13** does not exist. Complex **INT14** is transformed to *Z*-**P1** via **TS19**. Meanwhile, this pathway has a very high activation energy of 72.2 kcal/mol, thus, it may be excluded.

*Pathway (iiib).* The proton transfer may be directly assisted by the Pd atom to give the intermediate **INT15**. However, the energy of the latter is too high (69.4 kcal/mol) to permit the realization of this route.

*Pathway (iiic).* The coordination of Ind to the Pd center should increase the acidity of the NH group. This route includes the deprotonation of the ligated indazole by another free Ind molecule via **TS20** to give **INT16**. The cyclization of **INT16** to **INT17** via **TS21** and the protonation of the N isocyanide atom finally lead to *Z*-**P1**. The rate-limiting step of this route is the cyclization in **INT16** which occurs with a too high activation barrier (49.2 kcal/mol relative to **1**).

*Pathway (iv).* This pathway includes the addition of Ind to complex **1** to give the penta-coordinated intermediate **INT18**. However, the calculations indicated that such an intermediate does not exist. All attempts of its geometry optimization led to the extrusion of Ind to the second coordination sphere. These results are in agreement with those obtained previously for the substitution of the nitrile ligand in complexes *trans*-[MCl_2_(NCMe)_2_] (M = Pd, Pt) [93]. Therefore, all pathways considered in this part are not feasible either because the key intermediates do not exist or due to the very high activation barrier.

### 3.5. Bond Analysis

Previously [94,95], some of us demonstrated that the coordination of isocyanides to a metal center plays a tremendous role in the activation of C≡NR toward NA. Therefore, in this section we discuss results of the theoretical analysis of the coordination Pd–C bond nature in the starting Pd isocyanide complex **1**, in two isomeric products *Z*-**P1**, and *Z*-**P2** and in two rate-determining transition states leading to these products, **TS6** and **TS14**. Additionally, the nature of the CN bond between **1** and Ind fragments in **TS6** and **TS14** was also analyzed.

**Figure 2:**
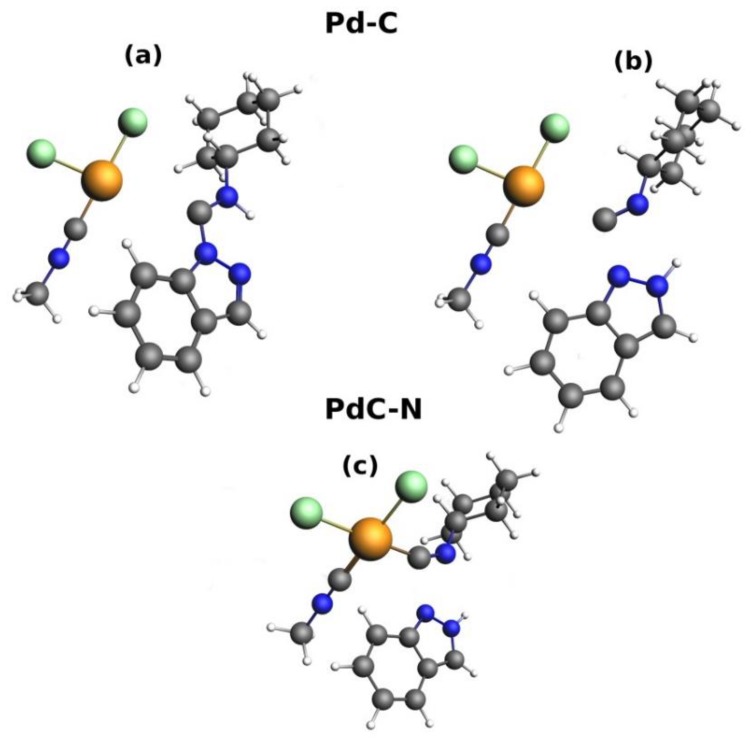
Schematic depictions of the fragments considered for the Pd–C interaction in *Z*-**P1** (**a**) and **TS14** (**b**) and for the PdC–N_Ind_ interaction in **TS14** (**c**). Analogous fragmentation schemes were used for **1** (**a**), *Z*-**P2** (**a**), and **TS6** (**b,c**).

The interaction between the {(MeN≡C)(Cl)_2_Pd} and carbene fragments was considered for the analysis of the Pd–C coordination bond, whereas the interaction between {Ind} and {**1**} with unrelaxed geometries corresponding to **TS6** or **TS14** were analyzed for the investigation of the PdC–N_Ind_ bond properties. The fragmentation of the model systems in *Z*-**P1** and **TS14** is shown in Figure 2.

**Figure 3:**
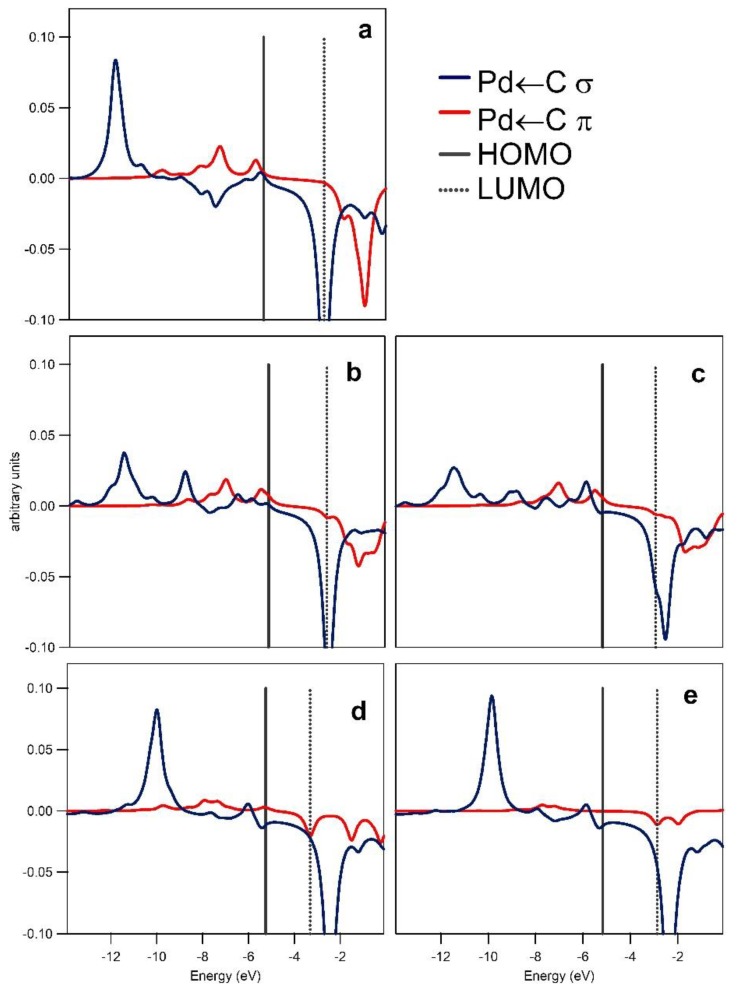
Crystal orbital overlap populations (COOPs) for the Pd–C interaction in **1** (**a**), **TS6** (**b**), **TS14** (**c**), *Z*-**P2** (**d**), and *Z*-**P1** (**e**). Bonding/antibonding interactions correspond to the positive/negative peaks, respectively. Solid and dotted vertical lines correspond to the highest occupied molecular orbital (HOMO) and the lowest unoccupied molecular orbital (LUMO), respectively.

**Figure 4:**
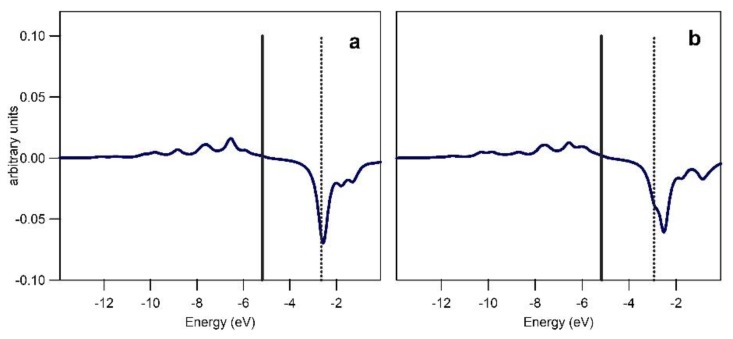
COOPs for the PdC–N interaction in **TS14** (**a**) and **TS6** (**b**). Solid and dotted vertical lines correspond to the HOMO and LUMO, respectively.

Crystal orbital overlap population (COOP) analysis and inspection of the relevant frontier molecular orbitals provided information on the Pd ← C (σ type; σ) and Pd → C (π type; π) interactions for the fragmentations (a) and (b) and on the PdC ← N (σ type; σ**′**) interactions for the fragmentation (c), see Figure 2. Overlap integrals for the interacting fragments and relevant populations are given in Table S1 in the Supplementary Material. Results of the COOP analysis depicted in a graphical form in Figure 3 and Figure 4 allow the interpretation of the bonding/anti-bonding nature of the discussed interactions. For the Pd–C bond, σ and π interactions show positive overlaps (bonding type) with the exceptions of the low anti-bonding σ interaction in the −8 to −5 eV energy range for **1**. The π interactions give a non-negligible contribution to the Pd–C bond as inferred from the EDA analysis (see Supplementary Material). Finally, the corresponding COOPs for the σ**′** interaction, see Figure 4, show very small overlap in agreement with the weak bonding between **1** and indazole in **TS6** and **TS14**. 

For all the models under study, the σ interactions mainly arise from the involvement of the C_CN_ electron pair essentially localized at orbitals with the C_2s_ and C_2px_ character and a virtual orbital with the Pd_dz2_, P_d5s_, Pd_d(x2-y2)_, and Cl_px_ characters. Moreover, the π interactions reveal the back-donation from orbitals with the predominant Pd_dxz/dxy_ + Cl_py_/_pz_ character, to the C_CN_ virtual orbitals of the p_z/y_ type. This effect is slightly more predominant in **1** compared to *Z*-**P1** and *Z*-**P2** (see VDD and EDA analyses in the Supplementary Material).

**Figure 5:**
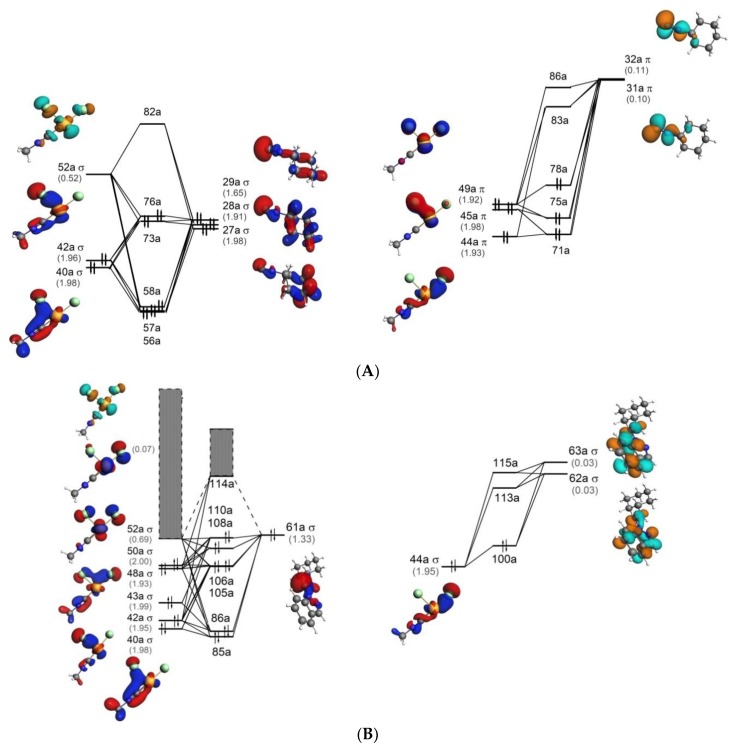

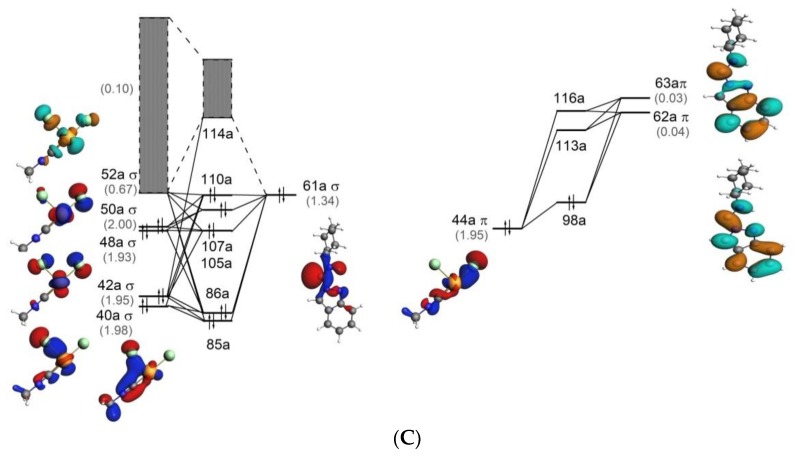
Molecular orbital diagrams for **1** (**A**), *Z*-**P1** (**B**), and *Z*-**P2** (**C**) for the Pd ← C (σ type) (left) and Pd → C (π type) (right) interactions. Corresponding orbitals populations (in e) are given in parentheses. Isosurface values are 0.05 e^1/2^/Å^3/2^. Gray bands indicate the overall participation of the virtual orbitals with populations other than 0.00.

Molecular orbital (MO) diagrams for **1**, *Z*-**P1**, and *Z*-**P2** are shown in Figure 5. These diagrams demonstrate the relative energies of MOs of these structures and their composition in terms of the orbital interaction of the fragments shown in Figure 2. Comparison of **1** with *Z*-**P1** and *Z*-**P2**, see Figure 5, shows appreciable differences in the MO composition. In particular, several σ highest occupied molecular orbitals (HOMOs) of *Z*-**P1** and *Z*-**P2** are composed by only one molecular orbital of the carbene ligand (61a), while three orbitals of C≡NCy (27a–29a) are involved in the σ bonding in **1**.

The different bonding mechanisms characterizing the reactant and the product(s) implies a stronger Pd–C bond energy of both *Z*-**P1** and *Z*-**P2** of about 15 kcal/mol with respect to **1** (the detailed discussion of the EDA and VVD analyses is provided in Supplementary Material) determining a further stabilization of the Pd–C bond and, thus, of the newly formed carbene.

**Figure 6:**
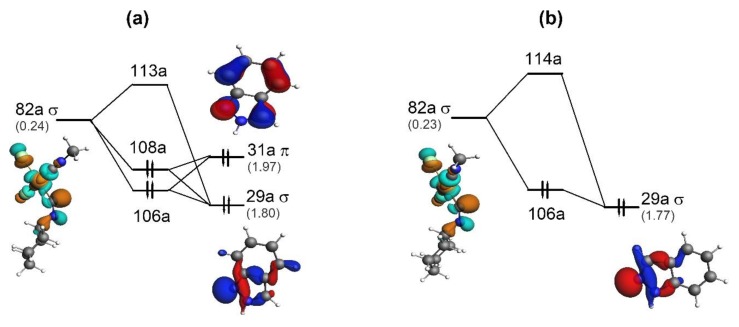
Molecular orbital diagrams for the PdC ← N (σ-type) interaction in **TS14** (**a**) and **TS6** (**b**). Corresponding orbitals populations (in e) are given in parentheses. Isosurface values are 0.05 e^1/2^/Å^3/2^.

Finally, the bonding schemes illustrating the PdC ← N interactions in **TS6** and **TS14** are given in Figure 6. In contrast to **TS6**, the orbital 31a of indazole participates in the σ′ interaction in **TS14**. Despite the 31a orbital of indazole showing π character, the orientation of the interacting fragments provides the σ-type interaction between {Ind} and {**1**}.

## 4. Final Remarks

Mechanisms of the addition of *N*-nucleophiles to metal-bound isocyanides—a promising and efficient route toward practically important *N*-heteroatom stabilized carbenes—are still poorly explored. In this work, the mechanism of the reaction between indazole and the Pd-coordinated cyclohexyl isocyanide in the model complex *cis*-[PdCl_2_(CNMe)(CNCy)] (**1**) was investigated in detail by DFT (M06L) methods.

The amino N(H) atom of Ind was found to be inactive toward nucleophilic addition. Instead, indazole reacts with **1** by the unprotonated nitrogen atom. Therefore, the mechanism of this reaction is different from that previously established for NA of amines, imines, and hydrazones to Pt-bound isocyanides [64].

The mechanism of reaction between Ind and **1** includes (*i*) nucleophilic attack of Ind by the unprotonated N atom at the isocyanide C atom, (*ii*) deprotonation of the resulting intermediate by another Ind molecule, and (*iii*) protonation of the isocyanide N atom to give the carbene product.

Two reaction channels based on different tautomeric forms of Ind were found. The first channel is based on the less stable tautomer of Ind and it leads to the experimentally isolated aminocarbene product **P1** (Mechanism III, Scheme 8). The second channel results in the formation of the isomeric carbene product **P2** which was not detected experimentally and is based on the most stable tautomer of Ind (Mechanism II, Scheme 6).

The second channel is slightly more favorable kinetically compared to the first one (by 2.5 kcal/mol). However, **P1** is thermodynamically more stable than **P2** (by 7.3 kcal/mol), the latter being endergonic relative to the reactants by 3.6 kcal/mol. Thus, the regioselectivity of this reaction is thermodynamically rather than kinetically driven. The nucleophilic addition of Ind to **1** by the amino N atom as well as the isomerization of **P2** into **P1** are not feasible.

The bonding nature in **1**, *Z*-**P1**, *Z*-**P2**, and the rate-limiting **TS6** and **TS14** were analyzed in detail.

## Supplementary Materials

The following are available online, additional computational details and bond analysis discussion, Figures S1, S2, S4, S5, S8, S9: bonding schemes, Figure S3: VDD charges, Figure S6: optimized structures of **R1** and **RP**, Figure S7: COOPs for **R1** and **RP**, Figures S10–S14: energy profiles, Tables S1, S3, S5: VDD charges, Table S2: results of the EDA analysis, Tables S4 and S6: overlaps and relevant orbital populations, Table S7: calculated total energies, enthalpies, Gibbs free energies, and entropies, Table S8: Cartesian atomic coordinates of the equilibrium structures.
