# Supplementary material for: Reaction between Indazole and Pd-Bound Isocyanides—A Theoretical Mechanistic Study
Source: Molecules. 2018 Nov 10;23(11):2942. doi: 10.3390/molecules23112942 (PMC6278333; doi:10.3390/molecules23112942)
Supplement: Supplementary file 1 [file molecules-23-02942-s001.pdf]

Reaction between Indazole and Pd-Bound Isocyanides – a Theoretical Mechanistic Study

Girolamo Casella [1,2,†], Maurizio Casarin [3], Vadim Yu. Kukushkin [4] and Maxim L. Kuznetsov [4,5,*]

[1] Dipartimento di Scienze della Terra e del Mare, Università degli Studi di Palermo, Via Archirafi,22, 90123, Palermo, Italia; girolamo.casella@unipa.it (G.C.)
[2] Consorzio Interuniversitario di Ricerca in Chimica dei Metalli nei Sistemi Biologici (C.I.R.C.M.S.B.), Piazza Umberto I, 70121 Bari, Italia; girolamo.casella@unipa.it (G.C.)
[3] Dipartimento di Scienze Chimiche, Università di Padova, via F. Marzolo 1, 35131, Padova, Italia; maurizio.casarin@unipd.it (M.C.)
[4] International Group on Organometallic Chemistry, Institute of Chemistry, Saint Petersburg State University, Universitetskaya Nab., 7/9, Saint Petersburg 199034, Russia; v.kukushkin@spbu.ru (V.Y.K)
[5] Centro de Química Estrutural, Instituto Superior Técnico, Universidade de Lisboa, Av. Rovisco Pais, Lisbon 1049-001, Portugal; max@mail.ist.utl.pt (M.L.K.)
* Correspondence: max@mail.ist.utl.pt (M.L.K.); Tel.: +351-218-419-236
† G.C. performed this work during his leave of absence period at the Dipartimento di Scienze Chimiche, Università di Padova, Padova, Italia at the Prof. Maurizio Casarin group.

Supplementary Material

Computational Details

Energy Decomposition Analysis (EDA)

The corresponding bonding energies of the interactions between the fragments shown in Figure 2 were calculated by considering their decomposition into strain and interaction energy contributions according to the Ziegler-Rauk works as implemented in the ADF package [S1–S3]. According to this scheme, the total energy ΔE may be written as:

$$\Delta E = \Delta E_{int} + \Delta E_{strain} \quad (1)$$

$$\Delta E_{int} = \Delta E_{elec} + \Delta E_{Pauli} + \Delta E_{orb} + \Delta E_{disp} \quad (2)$$

where ΔE_{elec}, ΔE_{Pauli}, and ΔE_{orb} represent contributions due to the quasi-classical electrostatic interaction, the Pauli repulsion, and the orbital interaction, respectively. ΔE_{disp} represents the contribution due to dispersion corrections. Moreover, the last term ΔE_{strain} provides information about the energy required to distort structure of free fragments to the geometry they assume in the final complex. Finally, ΔE_{int} and $-\Delta E$ are defined as the bonding (BE) and dissociation (DE) energies, respectively.

Fragment approach does not implement the solvent effects. In this context, as a better approximation to describe also the solvent effect, the single fragments SCFs were run accounting for the solvent before to run the final SCF without inclusion of solvent. This allowed to combine the fragments with the appropriate orbitals energies due to the effect of the solvent.

Voronoi Deformation Density (VDD) analysis

The charge distribution has been determined by analysis of the electron density deformation using the Voronoi Deformation Density (VDD) approach [S4]. VDD atomic charges were calculated as:

$$\Delta Q_A = -\int_V \left(\rho(r) - \sum_i \rho_i(r)\right) dr \quad (3)$$

where V corresponds to the Voronoi cell of atom A into the molecule, ρ(r) is the electron density of the molecule, and $\Sigma\rho_i(r)$ is the promolecule, used as reference, obtained as superposition of non-interacting atomic densities. Thus, VDD charges represent the electron density flowed from/towards an atom. Positive and negative values correspond to loss or gain of electrons, respectively.

Crystal orbital overlap population (COOP)

Crystal orbital overlap population (COOP) [S5] was calculated as implemented in the ADF package.

Bond Analysis

*Bond analysis of **TS6** and **TS14***

Bonding schemes for **TS6** and **TS14** are reported in Figures S1 and S2. Transition states present an intermediate behavior between **1** and *Z*-**P1**, *Z*-**P2** where in the Pd bearing fragments are already involved almost all the orbitals occuring in the products, while the CN bearing fragment still show the orbitals involved in the Pd–C bonding in **1**.

VDD charges analysis

VDD charges (ΔQ) analysis for the fragment schemes in Figure 2 allowed to get further information about the electron density flow during the bond formation between the fragments.

Results are given in Table S1. Also in this case we start by discussing the model systems characterized by the Pd–C interaction.

We will consider the ΔQ values for Pd, Cl, C_{CN}, N_{CN} and the N_{Ind} (the latter is referred to the amino or imino nitrogen, for **TS14**/*Z*-**P1** and **TS6**/*Z*-**P2**, respectively). For all these systems, the trend for Pd and Cl is the same. The Pd–C bond formation causes an electron density depletion on Pd and an increasing of the electron density of one of the Cl atoms while the second Cl shows a small positive ΔQ value. For all the systems, the Cl atoms with negative ΔQ are in trans to the interacting fragments. Accounting for the bonding schemes discussed (Figures 5, S1, S2) this is in line with the composition of the **Pd**52a orbital, for which the electron density arising from the C_{CN} carbon to Pd in the σ interaction is localized into the more electronegative halogen atom. Concerning C_{CN}, despite its donating role in the σ interaction, its ΔQ value remains almost unchanged ranging from low negative values for **1**, **TS6** and **TS14**, to low positive values for *Z*-**P1** and *Z*-**P2**. In a qualitative way, this implies an almost similar contributions of the two Pd←C and Pd→C, donation and back-donation pathways. The effect of Pd–C interaction results very small for the N_{CN} and is quite similar to N_{ind}, considering both TSs and products.

Relevant ΔQ charges for σ' interactions in the TSs, are also reported in Figure S3. Interestingly, C_{CN} and N_{CN} show an opposite trend, with respect to σ interactions, by changing their ΔQ charge signs. This trend seems counter-intuitive with respect to an expected negative ΔQ charge on the C_{CN} ensued after the donation from the N_{Ind}. This could be explained accounting for a withdrawing effect of the N_{CN} atom which tends to polarize the increased electron density on the C_{CN} and further affects the ΔQ sign change for Pd and Cl, even if smaller in magnitude with respect to σ, with a cascade effect which causes a small electron density depletion on the halogens in favor of the Pd atom. Finally, the indazole hydrogens shows a small negative ΔQ charge likely attributable to the occurrence of a hydrogen bond between these atoms, if we considering the N_{CN}–H_{Ind} distances (see Figure S3), which are in the range for the occurrence of such interaction.

EDA analysis

More information about the energetic of the interactions so far discussed were provided by EDA analysis. The relevant energies contributions to the interactions, corresponding to the fragments schemes in Figure 2, are given in Table S2. Interestingly, accounting for the Pd–C interactions, it is possible to observe that the formation of both transition states leads to higher bonding energies (BE), around 20 kcal/mol, with respect to **1**. This is due either to the greater contribution provided by the attractive terms, (ΔE_{Elec} and ΔE_{Orb}), with respect to the repulsive ΔE_{Pauli} ones (increasing of ΔE_{Orb} and ΔE_{Pauli} are in accord with the bonding schemes in Figures S1 and S2), as well as, also to the non-negligible role due to the dispersion corrections. Nonetheless, the ΔE_{Int} values reconciles the expected behavior, *i.e.*, a lower stability of the TSs with respect to compound **1**, due to the high destabilizing ΔE_{prep} term, arising from the high distortion of the C≡N bond during the transition state formation. To note that we found that **TS6** resulted slightly more stable with respect to **TS14**, thus rendering *Z*-**P2** kinetically favorite. Nonetheless, the Pd–C bond of **TS14** resulted more stable mainly due to the contribution of the ΔE_{disp} term. Concerning **TS6** and **TS14**, the same stability trend was observed for the σ' interaction still mainly ruled by the dispersions corrections term.

Moving from the TSs to the products, as could be expected, the Pd–C bond resulted more stabilized, in *Z*-**P1** and *Z*-**P2** (Table S2). The ΔE_{Elec} and the ΔE_{Pauli} terms continue to increase according to the formation of the CNCyInd moiety, while the ΔE_{orb} terms remain almost unchanged. These latter results indicate that the change in the orbital interactions is already attained in the TS. The BE and the ΔE_{Int} of *Z*-**P1** resulted slightly more stable than *Z*-**P2**, and an inspection of Table S2 still indicates that ΔE_{disp} term is the discriminant contribution to the different stability of the two products.

Finally, due to the lack of symmetry in these systems, it was not possible to quantify the contribution of the σ and π interactions to the ΔE_{Orb} term. In this respect, we tried to estimate these

contributions by inspection of the Pd–C bond in homologous *cis*-$PdCl_2(CNMe)_2$ and *cis*-$PdCl_2$(CNMe)(CNPh) symmetric systems, in C_s symmetry, at least for what concerns the compound **1**. For both symmetric models, bonding mechanisms, and relevant orbital populations, along with EDA analysis resulted very similar to **1**, then allowing a comparison between these systems (bonding schemes, VDD, and EDA analyses are given in Figures S4, S5 and Table S3). To note that the π interactions were recognized in both A' and A'' irreducible representations (irreps) and they correspond to the Pd44a-L31a and Pd(45,49)a-L32a ones given in Figure 5, respectively. Interactions in the A' irreps were not determined due to the occurrence of the σ interactions in this irrep. However, only the ΔE_{Orb} terms corresponding only to a π interaction were estimated around to 14–15 kcal/mol from a total ΔE_{Orb} of 75–77 kcal/mol (thus, *ca.* 20 %), to which we should consider the unknown contribution arising from A' π. We cannot make an estimation for the products, due to the lack of any likely model showing symmetry; nonetheless, accounting for the corresponding COOPs (Figure 2), small π overlaps, and the bonding schemes (Figure 5) where only the analogous A' π interaction is given, this should indicate a minor contribution to the Pd–C bond, with respect to **1**.

Bond analysis of the real molecular systems

The model systems so far investigated were chosen as simplified paradigm of the *cis*-[$PdCl_2${C(Ind)vN(H)Cy}(CNCy)] (**RP**) complex obtained by reaction of the *cis*-[$PdCl_2(CNCy)_2$] (**R1**) with indazole (see Figure S6). Results for **R1** and **RP** were in very close agreement with what obtained for the corresponding model systems **1** and *Z*-**P1** so far discussed, which further supports the reliability of the proposed reaction mechanisms also for the real system. Relevant FMO overlaps, COOPs, bonding schemes, VDD and EDA analyses are given in Tables S4, S6 and Figures S7, S8).

References

S1. Ziegler, T.; Rauk, A. On the Calculation of Bonding Energies by the Hartree Fock Slater Method. *Theor. Chim. Acta* **1977**, *46*, 1–10.

S2. Ziegler, T.; Rauk, A. A Theoretical Study of the Ethylene-Metal Bond in Complexes between Copper(1+), Silver(1+), Gold(1+), Platinum(0) or Platinum(2+) and Ethylene, Based on the Hartree-Fock-Slater Transition-State Method. *Inorg. Chem.* **1979**, *18*, 1558–1565.

S3. Ziegler, T.; Rauk, A. Carbon Monoxide, Carbon Monosulfide, Molecular Nitrogen, Phosphorus Trifluoride, and Methyl Isocyanide as σ-Donors and π-Acceptors. A Theoretical Study by the Hartree-Fock-Slater Transition-State Method. *Inorg. Chem.* **1979**, *18*, 1755–1759.

S4. Fonseca Guerra, C.; Handgraaf, J.-W.; Baerends, E. J.; Bickelhaupt, F. M. Voronoi Deformation Density (VDD) Charges: Assessment of the Mulliken, Bader, Hirshfeld, Weinhold, and VDD Methods for Charge Analysis. *J. Comput. Chem.* **2004**, *25*, 189–210.

S5. Miller, G. J. Solids and Surfaces: A Chemist's View of Bonding in Extended Structures. R. Hoffmann. VCH Publishers, New York/VCH Verlagsgesellschaft, Weinheim **1988**. *Angew. Chem.* **1989**, *101*, 1570–1571.

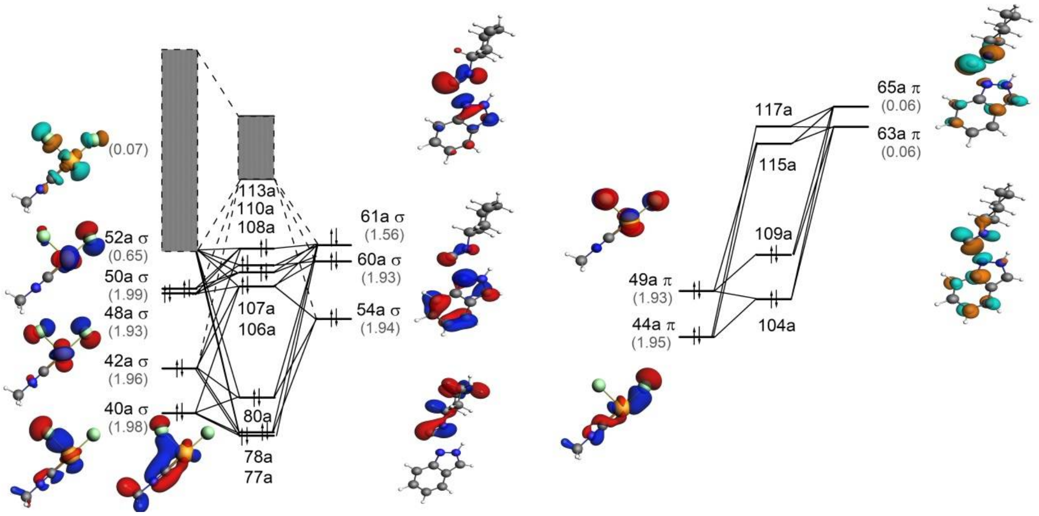

Figure S1. Bonding scheme for **TS14**. Left scheme: Pd←C (σ type). Right scheme: Pd→C (π type). Corresponding orbitals populations are given in parentheses. Isosurfaces values: 0.05 $e^{1/2}/Å^{3/2}$. Gray bands indicate the overall participation of the virtual orbitals with populations other than 0.00, and are mainly due to the polarization of the fragment bearing the Pd atom.

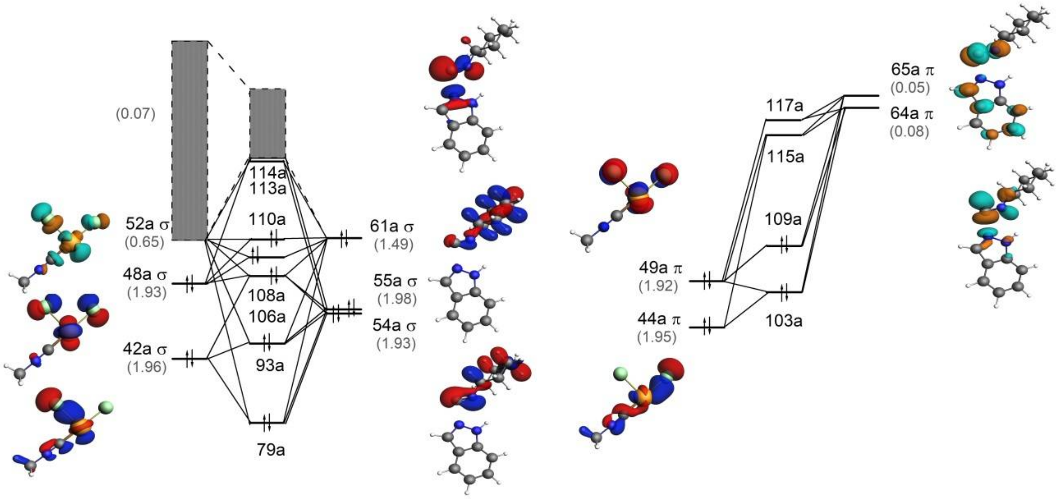

Figure S2. Bonding scheme for **TS6**. Left scheme: Pd←C (σ type). Right scheme: Pd→C (π type). Corresponding orbitals populations are given in parentheses. Isosurfaces values: 0.05 $e^{1/2}/Å^{3/2}$. Gray bands indicate the overall participation of the virtual orbitals with populations other than 0.00, and are mainly due to the polarization of the fragment bearing the Pd atom.

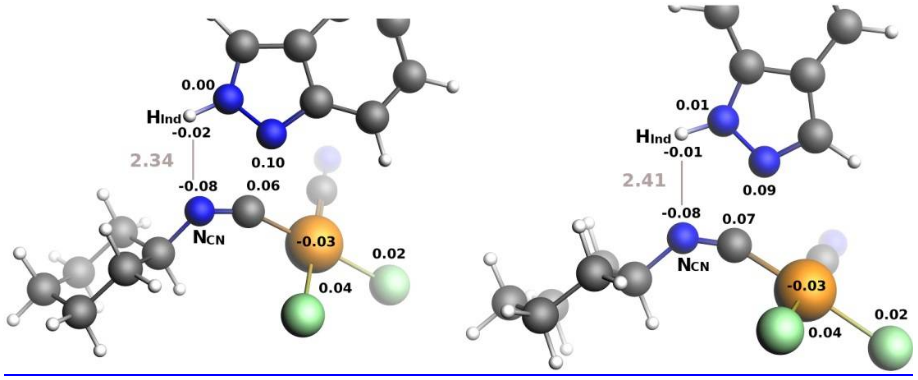

Figure S3. VDD charges (ΔQ), in electrons, of selected regions of **TS14** (left) and **TS6** (right) accounting for the σ' interaction (see fragment scheme in Figure 2). H_{Ind}–N_{CN} distances, in Angstrom, are given in gray.

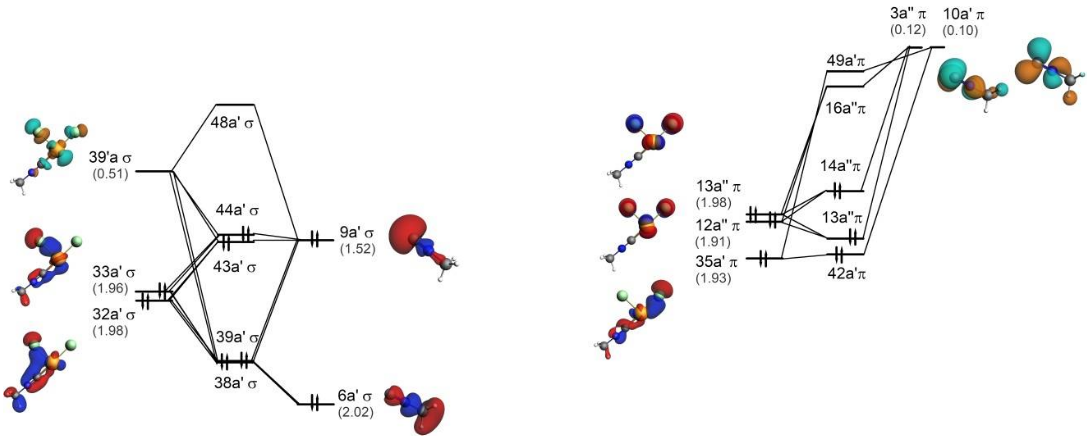

Figure S4. Bonding scheme for the symmetric *cis*-$PdCl_2(CNMe)_2$ model in C_s symmetry. Left scheme: Pd←C (σ type). Right scheme: Pd→C (π type). Corresponding orbitals populations are given in parentheses. Isosurfaces values: 0.05 $e^{1/2}/Å^{3/2}$. Gray bands indicate the overall participation of the virtual orbitals with populations other than 0.00, and are mainly due to the polarization of the fragment bearing the Pd atom.

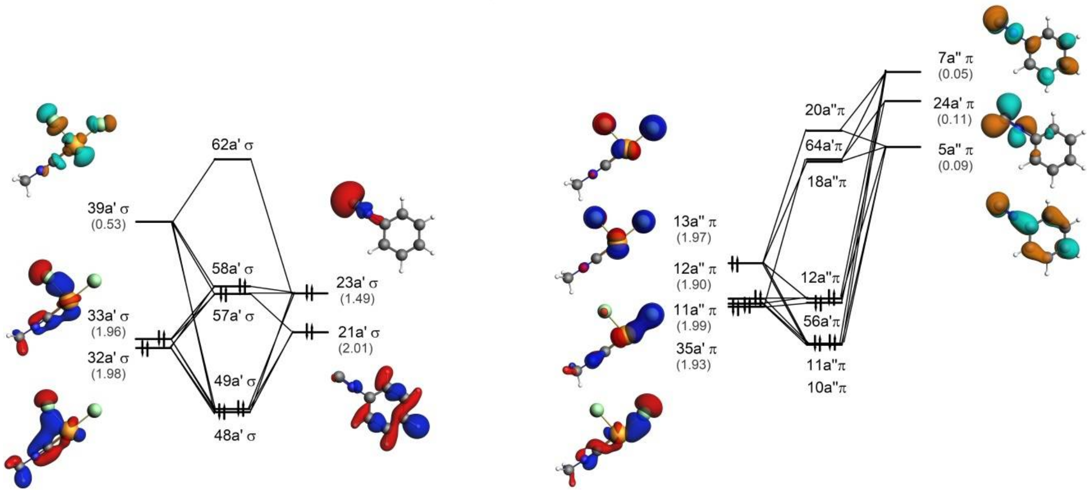

Figure S5. Bonding scheme for the symmetric *cis*-$PdCl_2(CNMe)(CNPh)$ model in C_s symmetry. Left scheme: Pd←C (σ type). Right scheme: Pd→C (π type). Corresponding orbitals populations are given in parentheses. Isosurfaces values: 0.05 $e^{1/2}/Å^{3/2}$. Gray bands indicate the overall participation of the virtual orbitals with populations other than 0.00, and are mainly due to the polarization of the fragment bearing the Pd atom.

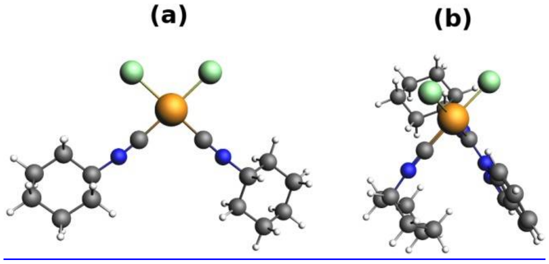

Figure S6. Optimized structures of *cis*-$[PdCl_2(CNCy)_2]$ (**R1**) (a) and *cis*-$[PdCl_2(CNCy)_2]$ (**RP**) (b).

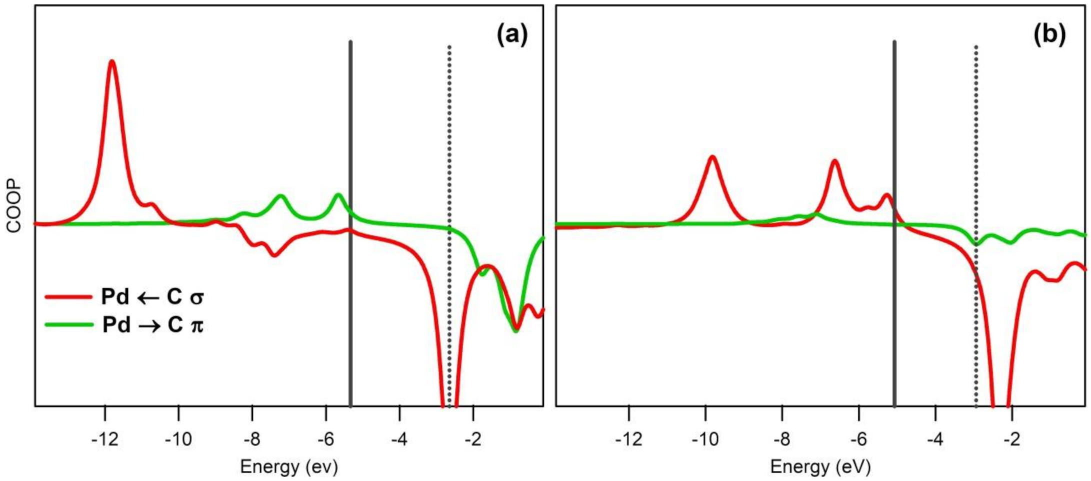

Figure S7. COOPs for **R1** (a) and **RP** (b) for the main bonding interactions. [Coordinates in arbitrary units. COOPs: x = –13.9, –0.1; y = –0.10, 0.12; PDOSs: x = –13.9, –0.1; y = –0.0, 2.0].

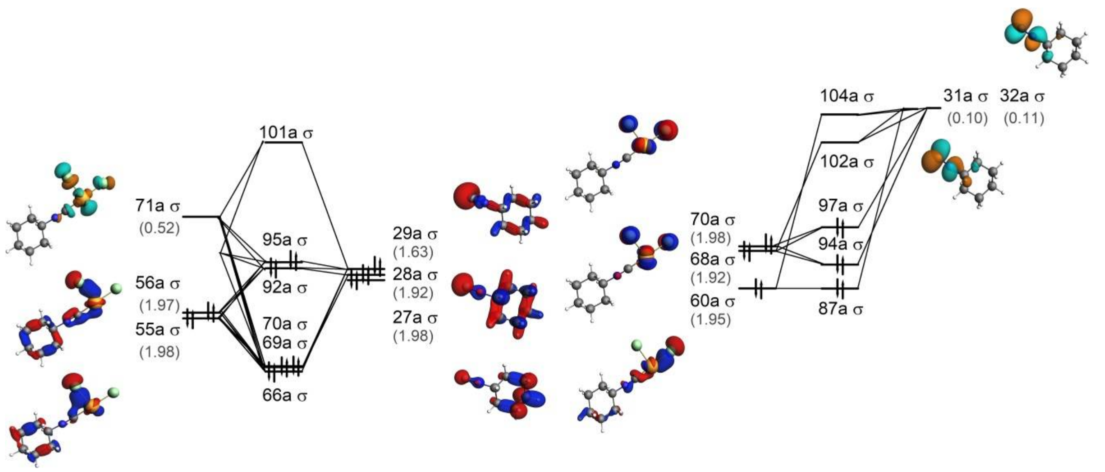

Figure S8. Bonding scheme for **R1**. Left scheme: Pd←C (σ type). Right scheme: Pd→C (π type). Corresponding orbitals populations are given in parentheses. Isosurfaces values: 0.05 $e^{1/2}/Å^{3/2}$. Gray bands indicate the overall participation of the virtual orbitals with populations other than 0.00, and are mainly due to the polarization of the fragment bearing the Pd atom.

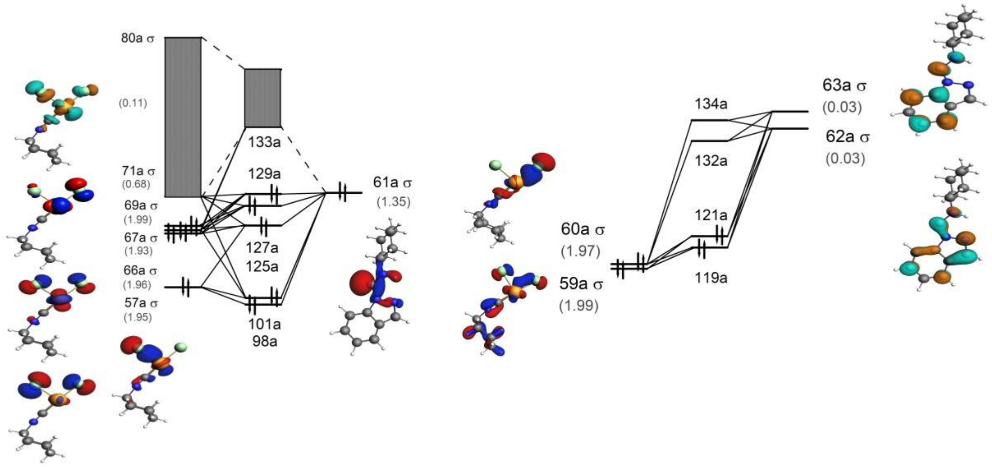

Figure S9. Bonding scheme for **RP**. Left scheme: Pd←C (σ type). Right scheme: Pd→C (π type). Corresponding orbitals populations are given in parentheses. Isosurfaces values: 0.05 $e^{1/2}/Å^{3/2}$. Gray bands indicate the overall participation of the virtual orbitals with populations other than 0.00, and are mainly due to the polarization of the fragment bearing the Pd atom.

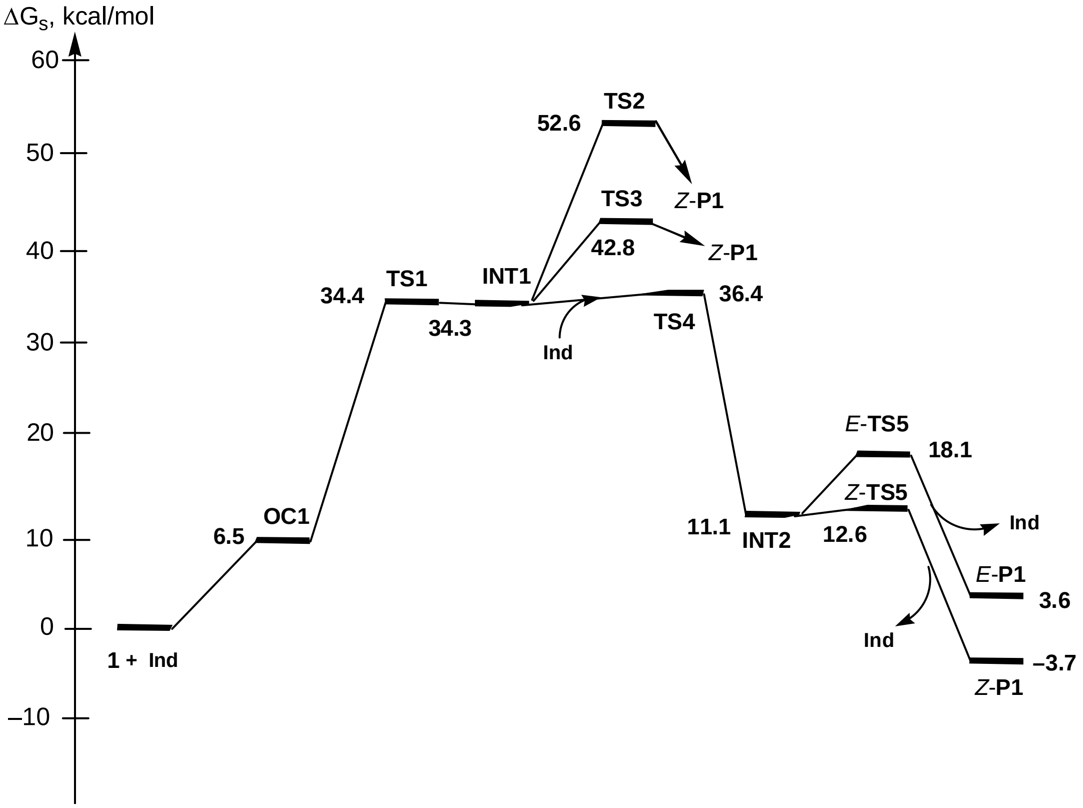

Figure S10. Energy profile for Mechanism I.

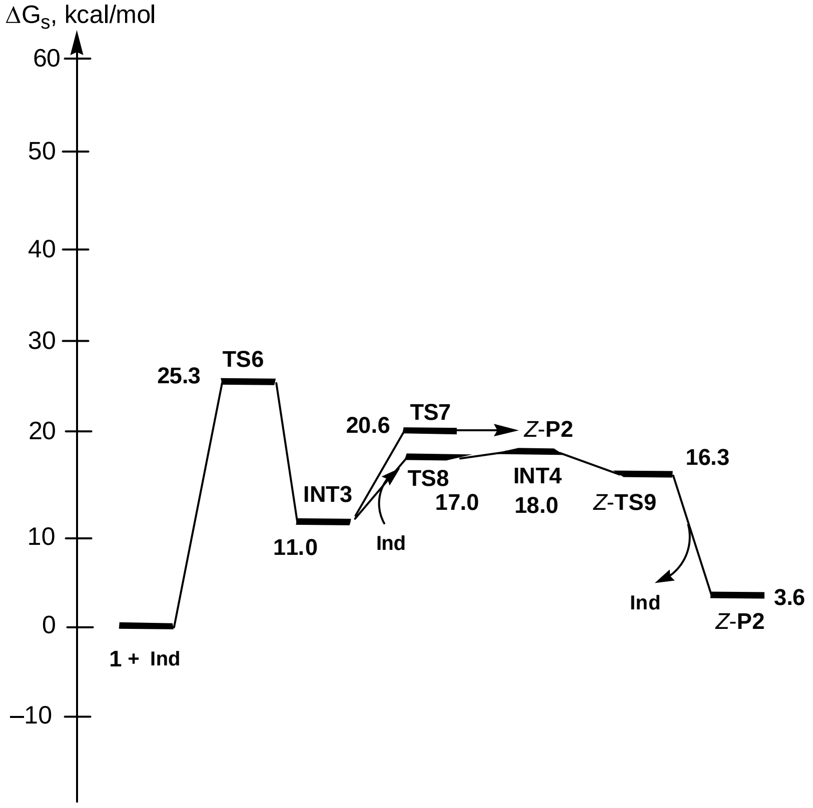

Figure S11. Energy profile for Mechanism II.

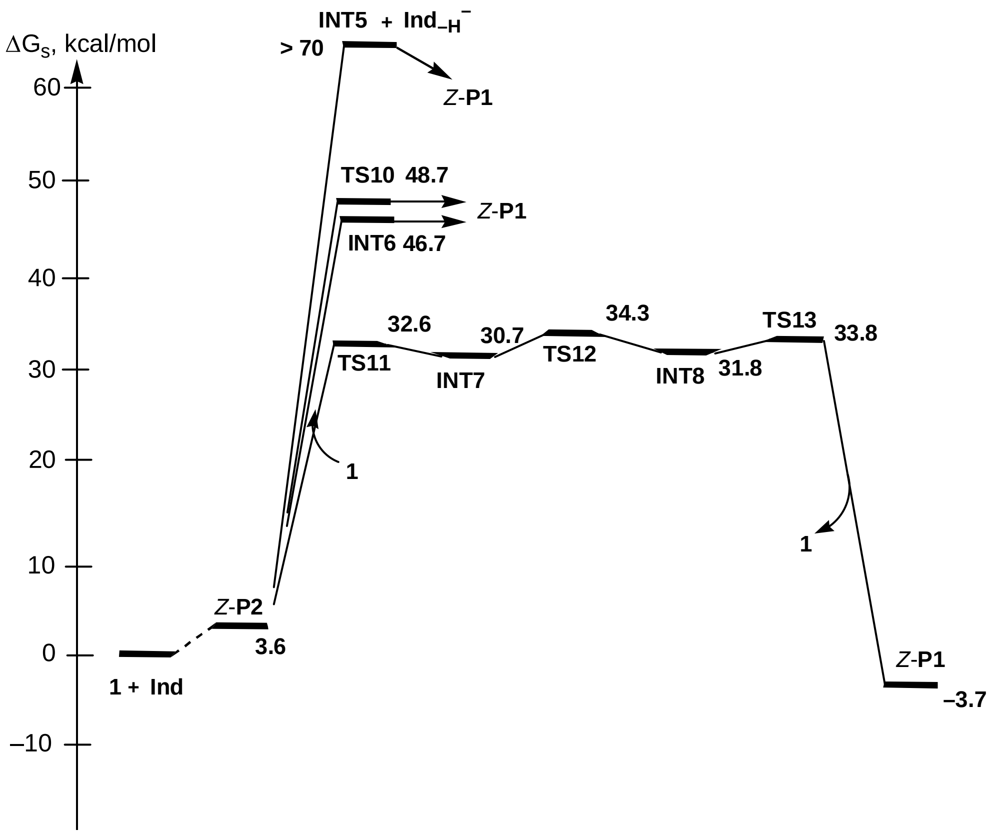

Figure S12. Energy profile for the **P2** into **P1** isomerization.

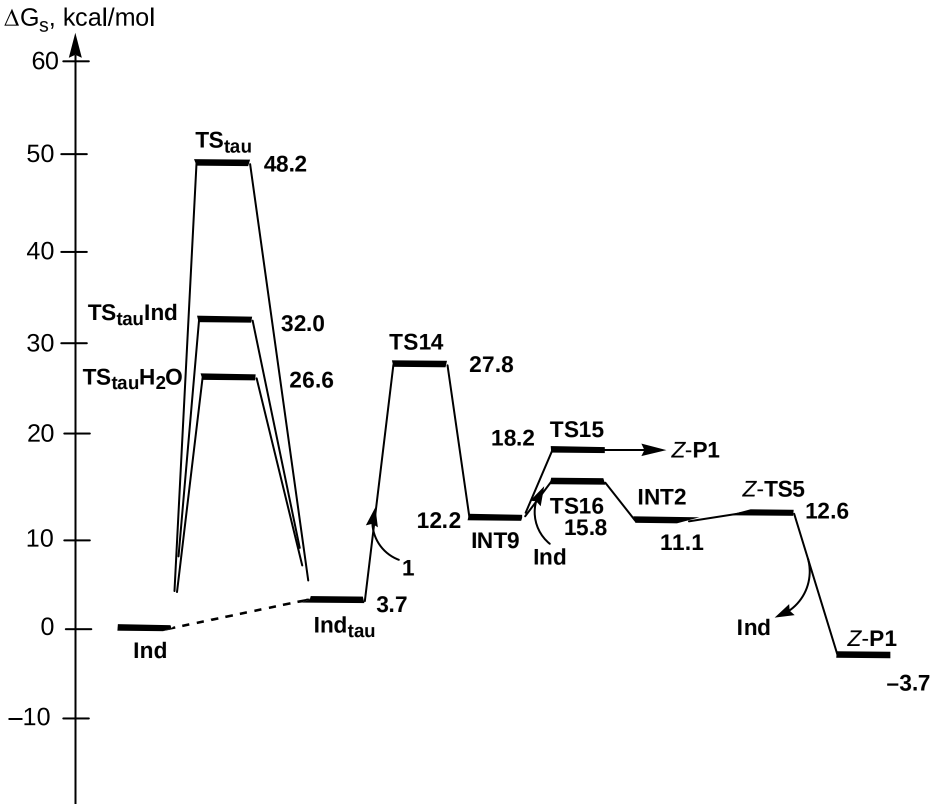

Figure S13. Energy profile for Mechanism III.

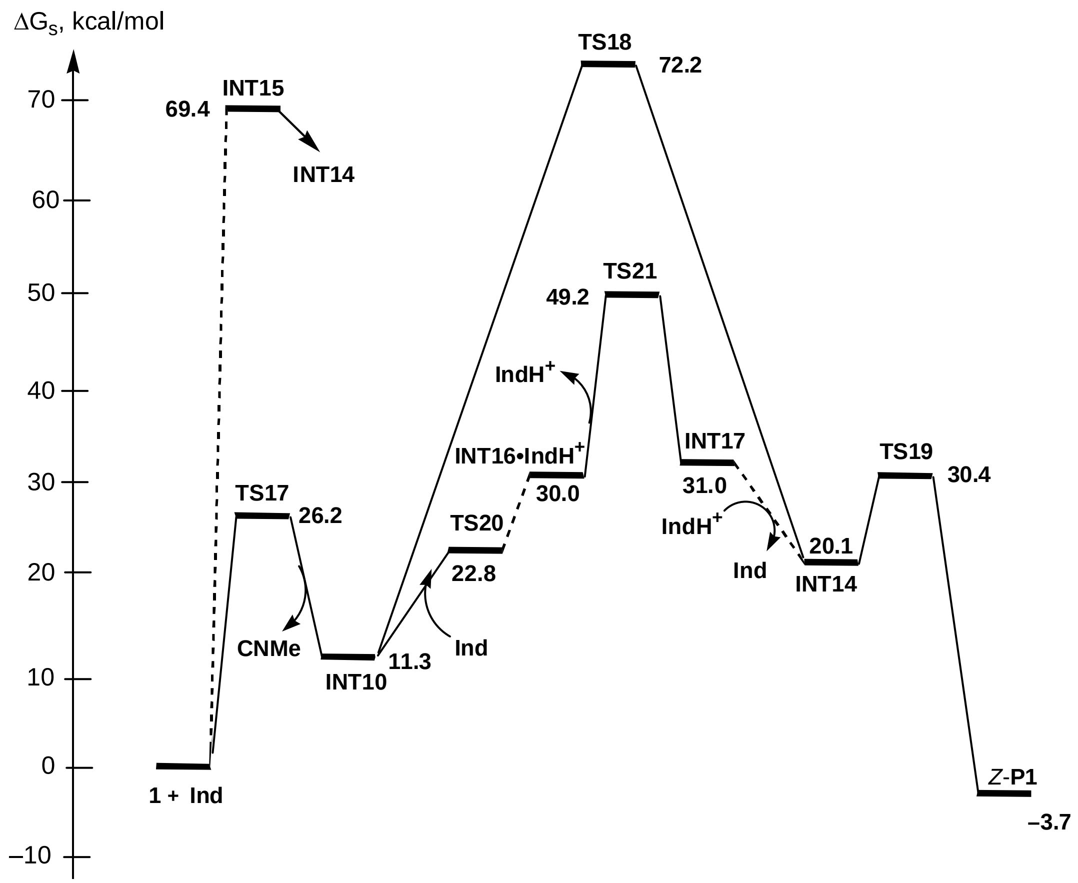

Figure S14. Energy profile for Mechanisms based on the coordinated indazole.

Table S1. VDD charges (ΔQ), in electrons, for the systems investigated.

	**1** (Pd–C)	**TS14** (Pd–C)	**TS6** (Pd–C)	**TS14** (PdC–N)	**TS6** (PdC–N)	***Z*-P1** (Pd–C)	***Z*-P2** (Pd–C)
ΔQ							
Pd	0.12	0.11	0.11	-0.03	-0.03	0.10	0.10
Cl	0.04; -0.10	0.02;-0.13	0.02;-0.13	0.02;0.04	0.02;0.04	0.01; -0.15	0.02;-0.14
C_{CN}	-0.01	-0.01	-0.01	0.06	0.07	0.02	0.01
N_{CN}	0.02	0.03	0.03	-0.08	-0.08	0.01	0.01
N_{Ind}	–	0.03	0.01	0.10	0.09	0.02	0.03

Table S2. EDA analysis. Contributions to the bonding energy (BE) and the interaction energy (ΔE_{int}) (kcal/mol) for the systems investigated according to the fragmentation scheme of Figure 2.

	**1** (Pd–C)	**TS14** (Pd–C)	**TS6** (Pd–C)	**TS14** (PdC–N)	**TS6** (PdC–N)	**ZP-1** (Pd–C)	**Z-P2** (Pd–C)
**EDA**							
ΔE_{Pauli}	176.38	196.74	194.81	86.96	82.56	221.08	217.60
ΔE_{Elec}	-154.43	-176.27	-176.56	-44.99	-42.88	-206.26	-204.15
ΔE_{Orb}	-72.77	-80.17	-78.70	-45.64	-43.00	-80.84	-78.85
ΔE_{disp}	-4.95	-13.84	-10.16	-11.76	-7.99	-15.57	-13.40
BSSE	-4.71	-6.87	-6.28	-5.88	-5.47	-6.16	-5.99
**BE**	**-60.48**	**-80.41**	**-77.33**	**-21.31**	**-16.78**	**-87.75**	**-84.79**
ΔE_{prep}	3.66	32.39	33.06	17.24	15.99	7.38	6.40
**ΔE_{int}**[a]	**-56.82**	**-48.27**	**-44.27**	**-4.1**	**-0.79**	**-80.37**	**-78.39**

[a] **ΔE_{int}= BE + ΔE_{prep}**

Table S3. VDD charges (ΔQ) (in electrons, e) and EDA analysis (kcal/mol) for the symmetric *cis*-$PdCl_2(CNMe)_2$ and *cis*-$PdCl_2$(CNMe)(CNPh). (Symmetry C_s)

	***cis*-$PdCl_2(CNMe)_2$ (Pd–C)**	***cis*-$PdCl_2(CNMe)_2$ (Pd–C)**
**ΔQ**		
**Pd**	0.13	0.13
**Cl**	0.04;-0.09	0.04;-0.09
**C_{CN}**	0.01	-0.04
**N_{CN}**	0.01	0.02
**EDA**		
ΔE_{Pauli}	181.38	183.07
ΔE_{Elec}	-156.82	-156.00
ΔE_{Orb}	-74.09	-77.24
$\Delta E^{A'}_{Orb}$	*-59.83*	*-61.67*
$\Delta E^{A''}_{Orb}$	*-14.27*	*-15.57*
ΔE_{disp}	-4.20	-4.75
BSSE	-5.41	-4.02
**BE**	**-59.85**	**-58.94**
ΔE_{prep}	1.61	1.67
**$\Delta E_{int}{}^{a}$**	**-58.24**	**-57.27**

[a] **ΔE_{int}= BE + ΔE_{prep}**

Table S4. Overlap ⟨L|Pd⟩ (L = CyNC; Pd = *cis*-$PdCl_2$) and relevant orbitals populations for the real systems investigated. Virtual orbitals are indicated with an asterisk.

	**Reactant (Pd–C)**	**Product (Pd–C)**
**FMO overlap, *S***		
	**Pd←C**	**Pd←C**
	0.05 ⟨28a|55a⟩	**0.15 ⟨61a|57a⟩**
	0.06 ⟨28a|56a⟩	0.06 ⟨61a|66a⟩
	0.03 ⟨28a|67a⟩	0.05 ⟨61a|67a⟩
	**0.15 ⟨28a|71a⟩**	**0.30 ⟨61a|71*a⟩**
	0.09	**0.16**

	⟨28a|73*a⟩	**⟨61a|73*a⟩**
	0.09<br>⟨29a|55a⟩	
	0.09<br>⟨29a|56a⟩	
	0.06<br>⟨29a|67a⟩	
	**0.29**<br>**⟨29a|71a⟩**	
	**0.15**<br>**⟨29a|73*a⟩**	
	**Pd→C**	**Pd→C**
	**0.13**<br>**⟨31*a|60a⟩**	0.04<br>⟨62*a|60a⟩
	0.10<br>⟨60a|32*a⟩	0.01<br>⟨62*a|59a⟩
	0.09<br>⟨68a|31*a⟩	0.02<br>⟨63*a|59a⟩
	**0.12**<br>**⟨68a|32*a⟩**	0.07<br>⟨63*a|60a⟩
	0.01<br>⟨70a|31*a⟩	
	0.02<br>⟨70a|32*a⟩	
**FMO population, e^-**		
**L**	28a(1.92)<br>29a(1.63)<br>31*a(0.10)<br>32*a(0.11)	61a(1.35)<br>62*a(0.03)<br>63*a(0.03)
**Pd**	55a(1.98)<br>56a(1.97)<br>60a(1.95)<br>67a(1.95)<br>68a(1.92)<br>70a(1.985)<br>71*a(0.52)<br>73*a(0.02)	57a(1.95)<br>59a(1.99)<br>60a(1.97)<br>66a(1.96)<br>67a(1.93)<br>71*a(0.68)<br>73*a(0.03)

Table S5. VDD charges (ΔQ) (in electrons, e) and EDA analysis (kcal/mol) for **R1**, and **RP**.

	**R1** (Pd–C)	**RP** (Pd–C)
**ΔQ**		
**Pd**	**0.12**	**0.10**
**Cl**	**0.04; -0.10**	**0.01;-0.15**
**C_{CN}**	**-0.01**	**0.01**
**N_{CN}**	**0.02**	**0.01**
**N_{Ind}**	**-**	**0.03**
**EDA**		
ΔE_{Pauli}	176.40	224.96
ΔE_{Elec}	-154.55	-206.76
ΔE_{Orb}	-72.77	-80.53
ΔE_{disp}	-4.95	-21.69
BSSE	-4.72	-5.65
**BE**	**-60.59**	**-89.67**
ΔE_{prep}	3.65	6.04
**ΔE_{int}[a]**	**-56.94**	**-83.63**

[a] $\Delta E_{int} = BE + \Delta E_{prep}$

Table S6. Overlap ⟨L|Pd⟩ (L = CyNC; CyNC); Pd = *cis*-$PdCl_2$) and relevant orbitals population of the systems investigated. Virtual orbitals are indicated with an asterisk.

	**1** (Pd–C)	**TS14** (Pd–C)	**TS6** (Pd–C)	**TS14** (PdC–N)	**TS6** (PdC–N)	*Z*-**P1** (Pd–C)	*Z*-**P2** (Pd–C)
**FMO overlap, *S***							
	**Pd←C**	**Pd←C**	**Pd←C**	**PdC←N**	**PdC←N**	**Pd←C**	**Pd←C**
	0.01 ⟨27a|40*a⟩	0.03 ⟨54a|40a⟩	0.07 ⟨54a|42a⟩	0.16 ⟨29a|82*a⟩	0.17 ⟨29a|82*a⟩	0.03 ⟨61a|40a⟩	0.03 ⟨61a|40a⟩
	0.02 ⟨28a|40a⟩	0.06 ⟨54a|42a⟩	0.03 ⟨54a|48a⟩	0.04 ⟨31a|82*a⟩		0.15 ⟨61a|42a⟩	0.16 ⟨61a|42a⟩
	0.05 ⟨29a|40a⟩	0.03 ⟨54a|48a⟩	0.14 ⟨54a|52*a⟩			0.04 ⟨61a|43a⟩	0.06 ⟨61a|48a⟩
	0.08 ⟨28a|42a⟩	0.01 ⟨54a|50a⟩	0.07 ⟨54a|54*a⟩			0.06 ⟨61a|48a⟩	0.10 ⟨61a|50a⟩
	0.12 ⟨29a|42a⟩	0.13 ⟨54a|52*a⟩	0.03 ⟨55a|42a⟩			0.10 ⟨61a|50a⟩	0.29 ⟨61a|52*a⟩
	0.09 ⟨27a|52*a⟩	0.01 ⟨60a|40a⟩	0.01 ⟨55a|48a⟩			0.31 ⟨61a|52*a⟩	
	0.16 ⟨28a|52*a⟩	0.05 ⟨60a|42a⟩	0.07 ⟨55a|52*a⟩				
	0.28 ⟨29a|52*a⟩	0.03 ⟨60a|48a⟩	0.11 ⟨61a|42a⟩				
		0.02 ⟨60a|50a⟩	0.05 ⟨61a|48a⟩				
		0.08 ⟨60a|52*a⟩	0.23 ⟨61a|52*a⟩				
		0.03 ⟨61a|4a⟩					
		0.10 ⟨61a|42a⟩					
		0.04 ⟨61a|48a⟩					
		0.06 ⟨61a|50a⟩					
		0.20 ⟨61a|52*a⟩					
	**Pd→C**	**Pd→C**	**Pd→C**			**Pd→C**	**Pd→C**
	0.08 ⟨31*|40a⟩	0.08 ⟨63*a|44a⟩	0.07 ⟨64*a|44a⟩			0.05 ⟨62*a|44a⟩	0.05 ⟨62*a|44a⟩
	0.15 ⟨31*|44a⟩	0.05 ⟨63*a|49a⟩	0.09 ⟨64*a|49a⟩			0.08 ⟨63*a|44a⟩	0.08 ⟨63*a|44a⟩
	0.02 ⟨31*|45a⟩	0.09 ⟨65*a|44a⟩	0.10 ⟨65*a|44a⟩				

	0.06<br>⟨31*|49a⟩	0.08<br>⟨65*a|49⟩	0.04<br>⟨65*a|49a⟩				
	0.04<br>⟨32*|40a⟩						
	0.08<br>⟨32*|44a⟩						
	0.05<br>⟨32*|45a⟩						
	0.13<br>⟨32*|49a⟩						
**FMO population, *e*[-]**							
**L**	27a(1.98)<br>28a(1.91)<br>29a(1.65)<br>31a*(0.10)<br>32a*(0.11)	54a(1.94)<br>60a(1.93)<br>61a(1.56)<br>63*a(0.06)<br>65*a(0.06)	54a(1.93)<br>55a(1.98)<br>61a(1.49)<br>64*a(0.08)<br>65*a(0.05)	29a(1.80)<br>31a(1.97)	29a(1.77)	61a(1.33)<br>62*a(0.03)<br>63*a(0.03)	61a(1.34)<br>62*a(0.04)<br>63*a(0.03)
**Pd**	40a(1.98)<br>42a(1.96)<br>45a(1.98)<br>44a(1.93)<br>49a(1.92)<br>52*a(0.52)	40a(1.98)<br>42a(1.96)<br>48a(1.93)<br>44a(1.95)<br>49a(1.93)<br>50a(1.99)<br>52*a(0.65)	42a(1.96)<br>44a(1.95)<br>48a(1.93)<br>49a(1.92)<br>52*a(0.65)	82*a(0.24)	82*a(0.23)	40a(1.98)<br>42a(1.95)<br>43a(1.99)<br>44a(1.95)<br>48a(1.93)<br>50a(2.00)<br>52*a(0.69)	40a(1.98)<br>42a(1.95)<br>44a(1.95)<br>48a(1.93)<br>50a(2.00)<br>52*a(0.67)

Table S7. Calculated total energies, enthalpies, Gibbs free energies (in Hartree) and entropies (in cal/mol•K).

	E (6-311+G**)	E (6-31G*)	H (6-31G*)	S	G (6-31G*)	G (6-311+G**)
**1**	–1509.428795	–1509.252574	–1509.011549	140.63	–1509.078366	–1509.254587
Ind	–379.905874	–379.810212	–379.684660	78.52	–379.721965	–379.817627
Ind (SMD in H_2O)	–379.902738	–379.807110	–379.681698	78.71	–379.719096	–379.814724
**Ind$_{tau}$**	–379.900199	–379.804309	–379.678784	78.00	–379.715845	–379.811735
**IndH$^+$**	–380.331363	–380.237197	–380.098182	79.93	–380.136159	–380.230325
**IndH$^+$** (SMD in H_2O)	–380.343148	–380.248944	–380.110251	80.41	–380.148457	–380.242661
**Ind$_{-H}^-$**	–379.399851	–379.296741	–379.185594	77.51	–379.222423	–379.325533
**Ind$_{-H}^-$** (SMD in H_2O)	–379.416663	–379.313596	–379.202186	77.45	–379.238986	–379.342053
**TS$_{Tau}$**	–379.823338	–379.724887	–379.605299	77.91	–379.642317	–379.740768
**TS$_{tau}$H$_2$O**	–609.241821	–609.037722	–608.835631	111.35	–608.888538	–609.092637
**TS$_{tau}$Ind**	–759.779347	–759.586322	–759.335888	116.47	–759.391228	–759.584253
Z-**P1**	–1889.366612	–1889.098386	–1888.728581	171.02	–1888.809839	–1889.078065
E-**P1**	–1889.358560	–1889.090801	–1888.720227	165.29	–1888.798761	–1889.066520
Z-**P2**	–1889.354721	–1889.085915	–1888.716190	171.59	–1888.797718	–1889.066524
**INT1**	–1889.305906	–1889.037134	–1888.668817	168.32	–1888.748792	–1889.017564
**INT2**	–2269.268985	–2268.905033	–2268.409410	208.00	–2268.508237	–2268.872189
**INT3**	–1889.342007	–1889.073125	–1888.703793	172.73	–1888.785863	–1889.054745
**INT4**	–2269.257956	–2268.894098	–2268.398076	208.91	–2268.497338	–2268.861196
**INT6**	–2269.213562	–2268.848651	–2268.352585	206.30	–2268.450603	–2268.815514
**INT7**	–3398.769387	–3398.324951	–3397.712011	255.51	–3397.833413	–3398.277849
**INT8**	–3398.767887	–3398.323593	–3397.711196	253.91	–3397.831835	–3398.276129
**INT9**	–1889.340908	–1889.071969	–1888.702792	170.38	–1888.783746	–1889.052685
**INT10**	-1756.579037	-1756.344026	-1756.027844	155.88	-1756.101906	-1756.336917
**INT14**	-1756.570028	-1756.336450	-1756.019199	147.59	-1756.089324	-1756.322902
**INT15**	-1756.480042	-1756.242803	-1755.932820	156.31	-1756.007088	-1756.244327
**INT16**	-1756.101665	-1755.865746	-1755.563594	155.08	-1755.637278	-1755.873197
**INT16•IndH$^+$**	-2136.476642	-2136.145114	-2135.702383	191.22	-2135.793237	-2136.124765
**INT17**	-1756.104486	-1755.870249	-1755.566984	148.12	-1755.637358	-1755.871595
**TS1**	–1889.304196	–1889.034983	–1888.667918	168.99	–1888.748210	–1889.017423
**TS2**	–1889.270374	–1888.999997	–1888.636620	171.44	–1888.718077	–1888.988454
**TS3**	–1965.754930	–1965.451128	–1965.060296	179.03	–1965.145358	–1965.449160
**TS4**	–2269.226357	–2268.862667	–2268.371470	203.39	–2268.468106	–2268.831796
Z-**TS5**	–2269.262278	–2268.898565	–2268.406978	208.38	–2268.505986	–2268.869699
E-**TS5**	–2269.256691	–2268.892988	–2268.400934	202.71	–2268.497248	–2268.860951
**TS6**	–1889.314610	–1889.044517	–1888.677635	177.10	–1888.761779	–1889.031872
**TS7**	–1889.323083	–1889.052784	–1888.688336	169.94	–1888.769077	–1889.039376

**TS8**	–2269.255848	–2268.891048	–2268.399311	207.59	–2268.497943	–2268.862743
Z-**TS9**	–2269.256534	–2268.892315	–2268.400682	208.21	–2268.499610	–2268.863829
**TS10**	–1889.280055	–1889.011697	–1888.645511	170.10	–1888.726332	–1888.994690
**TS11**	–3398.763054	–3398.318186	–3397.707022	259.00	–3397.830081	–3398.274949
**TS12**	–3398.760199	–3398.314939	–3397.706997	252.28	–3397.826862	–3398.272122
**TS13**	–3398.763609	–3398.318855	–3397.707353	254.34	–3397.828200	–3398.272954
**TS14**	–1889.311570	–1889.041513	–1888.674658	175.01	–1888.757810	–1889.027867
**TS15**	–1889.326268	–1889.055976	–1888.691467	171.26	–1888.772839	–1889.043131
**TS16**	–2269.258400	–2268.893782	–2268.402344	205.56	–2268.500010	–2268.864628
**TS17**	-1889.311200	-1889.040599	-1888.673845	181.20	-1888.759941	-1889.030542
**TS18**	-1756.479658	-1756.243854	-1755.932292	150.98	-1756.004028	-1756.239832
**TS19**	-1889.309769	-1889.041176	-1888.673078	172.86	-1888.755210	-1889.023803
**TS20**	-2136.486694	-2136.154909	-2135.715395	187.24	-2135.804360	-2136.136145
**TS21**	-1756.072507	-1755.837382	-1755.536812	148.77	-1755.607496	-1755.842621

Table S8. Cartesian atomic coordinates (in Å) of the calculated equilibrium structures (either element symbols or nuclear charges are given in the first column).

Ind

N	-2.056986	2.122101	1.865767
N	-3.270752	2.338159	1.297698
C	-3.017634	2.710090	0.051586
H	-2.017189	1.827020	2.831908
H	-3.842366	2.941813	-0.613343
C	-1.023291	2.347943	1.007086
C	-1.622278	2.742452	-0.215922
C	0.365091	2.250566	1.164986
C	-0.805239	3.049725	-1.317262
C	1.141451	2.559758	0.062183
C	0.566113	2.954951	-1.165662
H	-1.243457	3.354849	-2.266249
H	1.219699	3.187788	-2.004390
H	2.225461	2.496957	0.142367
H	0.811438	1.946386	2.109576

$IndH^+$

H	1.416073	1.382989	-0.436302
N	3.002557	0.247789	0.213629
N	2.415174	1.218439	-0.519217
C	3.307219	1.842842	-1.282290
H	2.452035	-0.321640	0.847709
H	3.001065	2.650279	-1.933366
C	4.328599	0.231808	-0.077132
C	4.557090	1.252776	-1.042202
C	5.357249	-0.581364	0.413140
C	5.859073	1.472012	-1.537460
C	6.617218	-0.338704	-0.093558
C	6.868989	0.671967	-1.054747
H	6.047346	2.248208	-2.274523
H	7.885571	0.811757	-1.413535
H	7.448924	-0.945471	0.257403
H	5.169320	-1.357191	1.149722

Ind_{-H}^-

N	-2.031023	2.100406	1.933949
N	-3.225696	2.331612	1.314910
C	-3.014206	2.712484	0.042571
H	-3.853726	2.941937	-0.611980
C	-1.059913	2.346923	1.014049
C	-1.633904	2.749496	-0.238210
C	0.340491	2.255392	1.151880
C	-0.800297	3.053786	-1.331333
C	1.138000	2.559696	0.062863
C	0.572348	2.957239	-1.173875
H	-1.222021	3.361566	-2.290730
H	1.232933	3.189224	-2.010187
H	2.223298	2.494229	0.151759
H	0.780967	1.949549	2.102756

Ind_{tau}

H	1.311987	-0.075062	0.578048
N	0.296865	1.661149	0.294026
N	1.414428	0.917956	0.407113
C	2.577477	1.584511	0.280510
H	3.531409	1.078955	0.354570
C	0.786416	2.903491	0.076877
C	2.221405	2.916479	0.057401
C	0.055011	4.096154	-0.117869
C	2.919696	4.125905	-0.156933
C	0.763027	5.257319	-0.324088
C	2.184529	5.274050	-0.343767
H	4.008342	4.147476	-0.173606
H	2.695937	6.220553	-0.511231
H	0.226185	6.192377	-0.477340
H	-1.033395	4.083465	-0.103140

TS_{tau}

7	0.222782	-2.182678	-0.221302
7	-1.241083	-2.082361	-0.249732
6	-1.569841	-0.782786	-0.178613
1	-0.628644	-2.614234	0.627280
1	-2.616149	-0.496510	-0.152448
6	0.661070	-0.895597	-0.137727
6	-0.424458	0.024149	-0.173339
6	1.996489	-0.446149	-0.075912
6	-0.178319	1.416009	-0.171648
6	2.207502	0.914058	-0.064850
6	1.130015	1.838162	-0.113666
1	-0.999943	2.128858	-0.207503
1	1.353259	2.903537	-0.103440
1	3.225227	1.298225	-0.020723
1	2.820324	-1.156134	-0.048477

$TS_{tau}H_2O$

N	-3.162286	-1.953875	-0.279540
N	-4.358060	-1.639842	-0.874711
C	-4.346747	-0.379403	-1.308040
H	-3.429372	-3.350553	0.718501
H	-5.215115	0.041433	-1.804548
C	-2.389533	-0.834750	-0.349449
C	-3.101752	0.210635	-1.008733
C	-1.077583	-0.627757	0.119632
C	-2.497005	1.467856	-1.202065
C	-0.509742	0.615243	-0.079052
C	-1.211524	1.656846	-0.733958
H	-3.031035	2.274445	-1.704235
H	-0.723076	2.620824	-0.867799
H	0.501999	0.804233	0.277710
H	-0.533806	-1.425820	0.624322
O	-3.819421	-4.285183	0.869594
H	-3.308450	-4.776262	0.167378

H	-4.886215	-4.100967	0.356777
O	-2.407666	-4.597907	-1.359972
O	-5.861695	-3.646127	-0.274672
H	-2.438375	-3.619253	-1.287905
H	-2.878984	-4.789285	-2.184509
H	-6.047786	-4.212532	-1.042187
H	-5.394404	-2.762094	-0.649499

TS_{tau}ind

N	-4.147404	-0.412601	0.669107
N	-5.089081	0.270983	1.395439
C	-4.671177	1.526115	1.571178
H	-4.781401	-1.606278	-0.844299
H	-5.283595	2.233041	2.125954
C	-3.126727	0.453569	0.396057
C	-3.413177	1.732360	0.958265
C	-1.933283	0.232894	-0.317125
C	-2.499866	2.790691	0.800409
C	-1.050337	1.289363	-0.457486
C	-1.330495	2.560731	0.095969
H	-2.705970	3.775171	1.222255
H	-0.610970	3.367721	-0.037027
H	-0.119223	1.144103	-1.005134
H	-1.711295	-0.746438	-0.745742
N	-3.984761	-3.061346	0.241656
N	-4.598290	-2.625398	-0.869884
C	-4.825207	-3.616138	-1.722441
H	-3.815842	-2.282912	0.913368
H	-5.314502	-3.432666	-2.668916
C	-3.794501	-4.396487	0.132380
C	-4.324438	-4.791971	-1.132173
C	-3.209620	-5.314965	1.015975
C	-4.263350	-6.144237	-1.524543
C	-3.170258	-6.628989	0.599229
C	-3.688429	-7.041917	-0.653420
H	-4.661848	-6.460840	-2.485469
H	-3.626391	-8.092945	-0.925579
H	-2.726013	-7.375781	1.253796
H	-2.811392	-5.000945	1.976849

1

N	-3.378362	-1.739081	0.656574
C	-2.504915	-1.324906	1.309477
Pd	-1.126464	-0.596719	2.466154
Cl	0.507706	0.310434	3.922079
Cl	-2.912743	-0.054737	3.926241
C	0.337978	-1.036057	1.271948
N	1.233559	-1.296758	0.573347
C	2.344111	-1.594638	-0.244281
C	-4.702253	-3.723735	0.208458
C	-5.975111	-4.216861	-0.469637
C	-4.556058	-2.216016	-0.001409

C	-7.202505	-3.461539	0.027142
C	-5.772464	-1.447117	0.518550
C	-7.037097	-1.958146	-0.160505
H	3.223365	-1.751430	0.386080
H	2.526220	-0.758598	-0.925121
H	2.137888	-2.500250	-0.820994
H	-4.417702	-2.007008	-1.072527
H	-5.631552	-0.373107	0.347895
H	-5.835849	-1.595684	1.606538
H	-4.747011	-3.919284	1.289693
H	-3.816772	-4.241646	-0.179856
H	-5.878691	-4.084534	-1.558831
H	-6.084474	-5.294693	-0.296938
H	-7.905235	-1.417821	0.236540
H	-6.991206	-1.724795	-1.236002
H	-8.102000	-3.813813	-0.493427
H	-7.354191	-3.678919	1.095931

E-**P1**

C	-2.271533	-3.277356	-2.511786
C	-1.354652	-3.615243	-3.681486
C	-0.044061	-2.842595	-3.590161
C	0.654649	-3.086914	-2.257336
C	-0.273257	-2.742865	-1.096574
C	-1.583626	-3.521899	-1.173132
N	0.372283	-3.056991	0.193649
C	0.822221	-2.287939	1.153805
N	0.984463	-0.940638	0.904665
N	1.399308	-0.483882	-0.346347
C	1.663559	0.790764	-0.187217
C	1.431862	1.240156	1.147567
C	0.992339	0.093868	1.837060
C	1.548988	2.463619	1.820338
C	1.215282	2.502135	3.164065
C	0.755804	1.351179	3.831232
C	0.624973	0.130674	3.182920
Pd	1.389472	-3.117240	2.903381
C	2.944984	-1.970476	2.971099
N	3.857253	-1.242652	3.006168
C	4.900582	-0.296909	3.072654
Cl	2.147188	-4.083640	4.976204
Cl	-0.498009	-4.566067	2.787317
H	0.302020	-4.037945	0.464896
H	4.622198	0.494766	3.775430
H	5.062126	0.134734	2.080269
H	5.817917	-0.783771	3.414490
H	2.026700	1.361317	-1.036047
H	1.886435	3.355927	1.297318
H	1.293424	3.438162	3.713051
H	0.485255	1.418147	4.882952
H	0.257483	-0.752100	3.702336

H	-0.482305	-1.667765	-1.099728
H	-2.234471	-3.239163	-0.335718
H	-1.368725	-4.596764	-1.057717
H	0.940841	-4.147647	-2.176279
H	1.573150	-2.494175	-2.173597
H	-0.246835	-1.765066	-3.698056
H	0.626848	-3.115011	-4.414847
H	-3.198135	-3.863049	-2.560390
H	-2.569818	-2.219075	-2.576473
H	-1.858254	-3.405050	-4.634084
H	-1.139918	-4.695730	-3.673709

***Z*-P1**

6	0.047477	2.420191	-0.611326
6	1.063841	3.476071	-0.191631
6	1.663588	3.151786	1.171729
6	2.280100	1.758042	1.194977
6	1.255710	0.713056	0.770762
6	0.662518	1.025595	-0.600834
7	1.881691	-0.612919	0.778142
6	1.314401	-1.719569	1.190498
7	2.080989	-2.853133	1.101740
7	3.355466	-2.765858	0.543345
6	3.852210	-3.975082	0.591486
6	2.951823	-4.913610	1.178714
6	1.805352	-4.163068	1.507928
6	3.010382	-6.283888	1.456750
6	1.913983	-6.871592	2.064845
6	0.780726	-6.109234	2.396002
6	0.701738	-4.748368	2.128511
46	-0.564607	-1.737191	1.880650
6	-1.259856	-2.295562	0.166907
7	-1.700772	-2.633066	-0.860991
6	-2.289413	-3.037591	-2.077098
17	-2.844872	-1.739750	2.681410
17	0.369613	-1.026409	3.959758
1	2.842136	-0.679098	0.433206
1	-1.710200	-3.858279	-2.509196
1	-2.302487	-2.194115	-2.773474
1	-3.313624	-3.371350	-1.889071
1	4.850036	-4.154594	0.204475
1	3.895573	-6.863018	1.202735
1	1.927310	-7.934096	2.297438
1	-0.062612	-6.592956	2.884464
1	-0.180541	-4.174395	2.406273
1	0.447620	0.673653	1.515644
1	-0.085554	0.261711	-0.857140
1	1.460589	0.961469	-1.358272
1	3.135941	1.719504	0.500543
1	2.665437	1.514133	2.193428
1	0.876888	3.207396	1.939883

1	2.420918	3.895509	1.450266
1	-0.355541	2.642547	-1.607685
1	-0.808005	2.440612	0.081992
1	0.597175	4.469584	-0.176348
1	1.869901	3.521932	-0.941803

***Z*-P2**

46	0.038331	-3.263237	1.430813
1	-2.854442	-0.720965	1.105548
17	1.383477	-5.257474	1.554094
17	-0.526267	-3.278389	3.745654
6	0.467810	-3.205241	-0.452298
7	0.738275	-3.202529	-1.588499
6	1.091286	-3.258964	-2.953050
1	1.881786	-4.001798	-3.090046
1	1.448179	-2.278295	-3.279684
1	0.215649	-3.543752	-3.543487
7	-1.290780	0.768903	1.377655
7	-0.521950	-0.361274	1.480554
6	0.797333	-0.114613	1.747972
1	1.493954	-0.936017	1.870467
6	-0.409776	1.757608	1.586972
6	0.921840	1.258720	1.826235
6	-0.662721	3.151275	1.589395
6	1.992884	2.153841	2.072663
6	0.398181	3.987056	1.829267
6	1.716667	3.495895	2.070873
1	2.997422	1.780348	2.259235
1	2.513687	4.212531	2.257461
1	0.236989	5.063599	1.837771
1	-1.665690	3.530681	1.407795
7	-2.377056	-1.627444	1.130789
6	-1.089512	-1.618309	1.329238
6	-4.490390	-2.720196	1.700735
6	-5.354825	-3.955701	1.472072
6	-3.184909	-2.834166	0.924040
6	-5.602609	-4.200317	-0.011620
6	-3.428144	-3.059895	-0.565630
6	-4.289961	-4.298853	-0.780049
1	-2.588184	-3.666108	1.326111
1	-2.463809	-3.153280	-1.084993
1	-3.932512	-2.173308	-0.982129
1	-5.029206	-1.820803	1.359307
1	-4.275981	-2.580957	2.768098
1	-4.849825	-4.831679	1.907316
1	-6.303584	-3.846043	2.012377
1	-4.476432	-4.440464	-1.852170
1	-3.733409	-5.186624	-0.440901
1	-6.196013	-5.112850	-0.152587
1	-6.201951	-3.371955	-0.422165

INT1

N	-0.242653	0.402705	0.704443
C	0.869972	0.401394	1.269389
Pd	2.209928	-0.946201	1.935268
N	1.415333	1.875630	1.540206
Cl	3.813110	-2.614840	2.686976
Cl	2.396440	0.340432	3.966546
C	2.105818	-1.955809	0.292618
N	2.102812	-2.605915	-0.678843
C	2.139118	-3.446901	-1.810616
C	-2.303462	-0.860129	1.014867
C	-3.056854	-2.126650	0.623901
C	-0.920905	-0.838425	0.367200
C	-3.152928	-2.278670	-0.890444
C	-1.033456	-0.950702	-1.151605
C	-1.773651	-2.225470	-1.538398
H	1.601728	-4.374153	-1.591071
H	3.179204	-3.681421	-2.054757
H	1.669502	-2.944748	-2.661780
N	0.366054	2.886168	1.565564
H	-0.350417	-1.707727	0.744202
H	-0.030145	-0.919954	-1.600079
H	-1.574126	-0.068937	-1.529547
H	-2.859116	0.031374	0.685361
H	-2.201290	-0.785596	2.105296
H	-2.534792	-3.001212	1.044091
H	-4.057212	-2.119807	1.075736
H	-1.854281	-2.297220	-2.631286
H	-1.183892	-3.097901	-1.211112
H	-3.662001	-3.216402	-1.149628
H	-3.772708	-1.464345	-1.298160
C	0.683922	3.741314	0.648303
H	1.803054	1.796439	2.509305
H	0.029169	4.592977	0.482439
C	2.405901	2.288093	0.557693
C	1.922744	3.452954	-0.036517
C	3.606583	1.698039	0.213576
C	2.655881	4.080636	-1.040948
C	4.334723	2.338047	-0.793052
C	3.867134	3.503067	-1.411392
H	2.297161	4.992334	-1.512260
H	4.467596	3.968305	-2.189749
H	5.290708	1.918324	-1.098087
H	3.968383	0.795015	0.702479

INT2

46	2.289624	-0.865069	-0.650017
1	-0.835610	-0.037329	-2.119602
17	4.310076	-1.644286	-1.813766
17	1.109006	-0.377704	-2.735536
6	3.144831	-1.163080	1.035284
7	3.694682	-1.346916	2.052225

6	4.381758	-1.596607	3.257140
1	3.887215	-1.069383	4.078321
1	5.414345	-1.246509	3.169497
1	4.380771	-2.670991	3.463389
7	-0.308773	-1.192269	0.616822
7	-0.189314	-2.433587	0.035346
6	-1.248057	-3.119038	0.404350
1	-1.367285	-4.139482	0.056627
6	-1.460240	-1.111826	1.390259
6	-2.106387	-2.361110	1.252505
6	-1.982071	-0.098730	2.202646
6	-3.331733	-2.594294	1.892779
6	-3.187561	-0.361370	2.837339
6	-3.865874	-1.584144	2.673713
1	-3.843083	-3.548512	1.779217
1	-4.817034	-1.736486	3.180221
1	-3.625264	0.406133	3.473946
1	-1.467622	0.850531	2.324260
7	-2.148423	1.001217	-0.875513
7	-1.835028	0.094466	-1.830014
6	-2.923781	-0.471511	-2.331095
1	-1.386075	1.258440	-0.211502
1	-2.842976	-1.187758	-3.138355
6	-3.496458	0.964604	-0.694550
6	-4.033528	0.046217	-1.637491
6	-4.304759	1.660182	0.213192
6	-5.422779	-0.179962	-1.685690
6	-5.660006	1.408429	0.146572
6	-6.215172	0.502982	-0.790107
1	-5.846637	-0.875677	-2.405522
1	-7.291565	0.348122	-0.796307
1	-6.326400	1.922263	0.836355
1	-3.880553	2.355960	0.932598
7	0.304032	1.014806	0.535034
6	0.634763	-0.198960	0.295601
6	0.608192	3.261546	-0.382673
6	1.578102	4.425368	-0.549623
6	1.276972	2.082019	0.317301
6	2.174591	4.850312	0.787642
6	1.870966	2.510325	1.659226
6	2.840960	3.672974	1.490085
1	2.102182	1.713222	-0.321535
1	2.367617	1.647141	2.126210
1	1.046618	2.801988	2.331122
1	-0.259987	3.581304	0.219907
1	0.215512	2.939232	-1.358233
1	2.389990	4.120696	-1.228393
1	1.072032	5.270461	-1.034695
1	3.237128	3.978856	2.467618
1	3.705220	3.337675	0.895216

1	2.893756	5.667478	0.643316
1	1.373988	5.249640	1.431533

INT3

46	1.301352	-2.929109	0.458917
1	-1.406326	0.653800	-0.338063
17	2.912994	-4.679397	1.005644
17	1.558188	-1.810021	2.558214
6	1.004515	-3.810928	-1.227638
7	0.795149	-4.380414	-2.227812
6	0.551496	-5.089722	-3.421221
1	0.206032	-4.398610	-4.195672
1	-0.215454	-5.849730	-3.243559
1	1.472900	-5.577392	-3.751923
7	-0.413364	0.871404	-0.305173
7	0.437621	-0.173032	-0.118459
6	1.694885	0.274384	-0.054641
1	2.506612	-0.425792	0.098177
6	0.299343	2.021192	-0.353687
6	1.670941	1.667879	-0.203455
6	-0.113507	3.349460	-0.515524
6	2.662272	2.669228	-0.222295
6	0.884037	4.304098	-0.526396
6	2.254077	3.973538	-0.384111
1	3.711368	2.409030	-0.107289
1	2.991661	4.772163	-0.400249
1	0.607360	5.349369	-0.646400
1	-1.162378	3.610951	-0.623587
7	-1.327284	-1.587935	-0.182408
6	-0.074191	-1.526070	-0.001465
6	-3.024850	-2.852398	1.035004
6	-3.784945	-4.172775	1.093774
6	-2.012167	-2.866567	-0.108862
6	-4.457756	-4.493818	-0.236257
6	-2.703938	-3.152181	-1.440352
6	-3.451362	-4.478727	-1.381963
1	-1.287161	-3.678224	0.090511
1	-1.961081	-3.152267	-2.251246
1	-3.406241	-2.331228	-1.656577
1	-3.725581	-2.017189	0.876437
1	-2.503612	-2.657180	1.981977
1	-3.080998	-4.980427	1.349353
1	-4.525504	-4.142081	1.903708
1	-3.951152	-4.673224	-2.340279
1	-2.724015	-5.295040	-1.238736
1	-4.963780	-5.466819	-0.183959
1	-5.241578	-3.745509	-0.436960

INT4

46	-2.583320	-0.644890	-0.493049
1	1.875428	1.960514	-0.653091
17	-4.707632	-1.615886	-1.254195

17	-2.104627	0.412038	-2.593988
6	-2.837876	-1.506564	1.205768
7	-2.961604	-2.083649	2.216996
6	-3.135374	-2.807458	3.413480
1	-4.160647	-3.184370	3.468055
1	-2.940174	-2.156271	4.270439
1	-2.437666	-3.651023	3.433943
7	0.335707	2.268989	0.298884
7	-0.819602	1.542669	0.299682
6	-1.926447	2.298954	0.498912
1	-2.908940	1.842193	0.518760
6	-0.074455	3.536080	0.510196
6	-1.497138	3.612946	0.650936
6	0.721828	4.700493	0.589369
6	-2.127544	4.856895	0.881338
6	0.079228	5.895602	0.812885
6	-1.333648	5.976483	0.957576
1	-3.208384	4.922129	0.988437
1	-1.788370	6.949929	1.129492
1	0.663976	6.811456	0.881614
1	1.804152	4.643865	0.483601
7	0.288547	-0.481646	0.314700
6	-0.816066	0.123969	0.124720
6	1.210765	-2.279911	-1.082006
6	1.335431	-3.792087	-1.231397
6	0.362614	-1.927817	0.140122
6	1.897916	-4.434426	0.031951
6	0.938467	-2.557750	1.406498
6	1.064369	-4.068495	1.255346
1	-0.648203	-2.341544	-0.028529
1	0.308080	-2.296715	2.268991
1	1.931098	-2.115540	1.601654
1	2.213269	-1.834371	-0.970329
1	0.761958	-1.830573	-1.978000
1	0.340490	-4.214937	-1.440684
1	1.961667	-4.032953	-2.100170
1	1.496309	-4.505719	2.165584
1	0.055888	-4.501720	1.151298
1	1.950443	-5.525058	-0.083181
1	2.933916	-4.088419	0.184028
1	1.984928	0.121899	0.600457
7	2.908869	0.534166	0.313340
7	2.806387	1.493133	-0.630708
6	3.959706	1.695685	-1.249948
1	4.055670	2.464832	-2.004345
6	4.171874	0.034535	0.260305
6	4.887792	0.779608	-0.720148
6	4.773036	-1.000305	0.989707
6	6.244399	0.495137	-0.968873
6	6.100800	-1.258955	0.714904

6	6.830897	-0.521929	-0.249072
1	6.802403	1.061751	-1.710439
1	7.875638	-0.770674	-0.420003
1	6.605694	-2.055133	1.257789
1	4.220932	-1.562945	1.738331

INT6

46	-0.024764	-1.046230	1.572489
1	-0.377590	-0.946176	-2.316311
17	0.645005	-1.404764	3.842624
17	-2.038484	-2.320505	1.810312
6	1.642375	-0.091586	1.320170
7	2.669054	0.438353	1.160672
6	3.896945	1.101304	0.958258
1	4.072941	1.797572	1.783320
1	3.859716	1.659858	0.015577
1	4.706066	0.364681	0.925242
7	-2.630574	-0.548550	-1.659589
7	-1.867436	-0.222546	-0.540091
6	-2.475291	0.712535	0.195279
1	-1.917958	1.195012	1.006228
6	-3.821525	0.128156	-1.518784
6	-3.723137	0.975800	-0.386327
6	-4.967285	0.075456	-2.310410
6	-4.789049	1.830952	-0.045923
6	-6.005792	0.914168	-1.941992
6	-5.919793	1.784472	-0.831327
1	-4.711922	2.494001	0.811803
1	-6.767799	2.422507	-0.595309
1	-6.919630	0.906549	-2.532197
1	-5.038679	-0.585609	-3.169810
7	0.005258	-1.195551	-1.400778
6	-0.587849	-0.759433	-0.319958
6	1.077771	-3.263262	-2.161349
6	2.392592	-4.035294	-2.208156
6	1.272007	-1.936905	-1.432257
6	3.511709	-3.205108	-2.826165
6	2.366476	-1.093664	-2.075675
6	3.675613	-1.873869	-2.101177
1	1.526651	-2.139667	-0.383297
1	2.472244	-0.145518	-1.532462
1	2.063404	-0.836798	-3.103954
1	0.726758	-3.059916	-3.186191
1	0.295695	-3.848483	-1.660239
1	2.673868	-4.321943	-1.183239
1	2.249975	-4.971355	-2.762578
1	4.459926	-1.266595	-2.569964
1	4.001295	-2.060360	-1.065093
1	4.454104	-3.767262	-2.810147
1	3.281499	-3.013350	-3.886184
7	0.073480	2.013623	-0.274437

7	-0.219039	2.348632	1.021612
6	0.666415	3.239256	1.479614
1	0.584213	3.629882	2.490533
6	1.188074	2.715116	-0.613792
6	1.618006	3.535276	0.478333
6	1.906571	2.714566	-1.827515
6	2.753755	4.353769	0.346269
6	3.028333	3.519319	-1.929131
6	3.449522	4.335722	-0.851208
1	3.085257	4.989788	1.168244
1	4.331857	4.962715	-0.975223
1	3.597415	3.536415	-2.858558
1	1.578166	2.098360	-2.666379
1	-2.613041	-1.536345	-1.910640

INT7

N	-0.649509	-1.087914	-1.183854
N	0.694276	-1.249732	-0.807858
C	1.007320	-2.561692	-0.773149
H	2.028884	-2.859984	-0.567263
C	-1.147898	-2.354316	-1.337825
C	-0.120947	-3.303613	-1.101889
C	-2.449202	-2.743827	-1.673640
C	-0.380861	-4.685674	-1.223881
C	-2.677470	-4.102322	-1.775430
C	-1.660454	-5.064465	-1.554086
H	0.409213	-5.413181	-1.059138
H	-1.904669	-6.119186	-1.652403
H	-3.673550	-4.447605	-2.044561
H	-3.229307	-2.012030	-1.864705
Pd	-2.490653	-0.482518	1.027019
Cl	-3.569093	-1.044143	3.143482
Cl	-0.321890	-0.287905	2.029405
C	-4.250869	-0.561893	0.229932
N	-5.351608	-0.561223	-0.168028
C	-6.686673	-0.488434	-0.614698
H	-7.361476	-0.528231	0.245017
H	-6.900777	-1.327127	-1.283296
H	-6.838452	0.453699	-1.152772
N	-1.458128	1.068236	-1.391764
C	-1.538874	0.031457	-0.674919
C	-3.534641	2.145242	-2.042716
C	-4.350435	3.431470	-2.016770
C	-2.246525	2.287177	-1.224946
C	-4.628050	3.887422	-0.589274
C	-2.537622	2.741491	0.204570
C	-3.329154	4.043824	0.192450
H	-1.646974	3.078753	-1.705593
H	-1.597243	2.853703	0.761924
H	-3.119766	1.971807	0.732430
H	-4.122402	1.319575	-1.614869

H	-3.285159	1.856383	-3.072207
H	-3.796628	4.221331	-2.548356
H	-5.287763	3.285962	-2.570525
H	-3.531752	4.363042	1.222505
H	-2.722927	4.841395	-0.267373
H	-5.193872	4.828140	-0.591144
H	-5.264268	3.139974	-0.085432
Pd	3.439878	-0.694980	0.063697
Cl	5.599262	-1.261840	0.934030
Cl	3.946157	-1.507404	-2.134385
C	2.999812	-0.103981	1.855413
N	2.798362	0.247182	2.948468
C	2.448055	0.671988	4.245508
H	3.354354	0.848441	4.831083
H	1.841163	-0.100410	4.726189
H	1.866450	1.596216	4.175092
N	1.320824	0.901625	-1.290682
C	1.658609	-0.217768	-0.726755
C	1.787376	2.851336	0.133176
C	2.365516	4.260347	0.154525
C	2.021637	2.204964	-1.230435
C	3.839165	4.258891	-0.234521
C	3.497943	2.169746	-1.602433
C	4.049999	3.592948	-1.589060
H	1.498793	2.802335	-1.991039
H	3.631900	1.693798	-2.581501
H	4.057546	1.561475	-0.875027
H	2.274584	2.231566	0.901216
H	0.713214	2.855599	0.366003
H	1.801692	4.891854	-0.550622
H	2.222926	4.702756	1.148468
H	5.115221	3.570836	-1.849350
H	3.550354	4.186122	-2.372016
H	4.231965	5.283719	-0.247290
H	4.415168	3.712018	0.528769
H	0.379723	0.974978	-1.717065

INT8

7	-0.740545	-1.184982	-1.068087
7	0.648954	-1.353742	-0.737543
6	0.889146	-2.651990	-0.564546
1	1.903620	-2.975825	-0.354433
6	-1.284474	-2.464273	-1.078804
6	-0.274267	-3.398002	-0.773788
6	-2.586112	-2.875209	-1.365832
6	-0.545290	-4.776810	-0.730666
6	-2.836824	-4.237563	-1.308712
6	-1.837294	-5.180262	-0.990698
1	0.245200	-5.486311	-0.501216
1	-2.093261	-6.236171	-0.964778
1	-3.841983	-4.589559	-1.530632

1	-3.365357	-2.174681	-1.646753
46	-3.063135	-0.282999	0.544225
17	-4.812549	-0.477961	2.178334
17	-1.306402	-0.724634	2.112186
6	-4.458914	0.026042	-0.765418
7	-5.322189	0.189623	-1.534867
6	-6.384187	0.410169	-2.436936
1	-7.330728	0.396527	-1.889595
1	-6.387990	-0.377444	-3.195833
1	-6.253584	1.382811	-2.920627
7	-1.020385	1.096080	-1.095262
6	-1.504514	-0.040164	-0.697022
6	-2.786575	2.899792	-1.210880
6	-2.980815	4.386134	-0.919343
6	-1.411303	2.469279	-0.715446
6	-2.808979	4.685373	0.565817
6	-1.264724	2.713755	0.785155
6	-1.459903	4.195641	1.079134
1	-0.661425	3.084550	-1.236304
1	-0.284026	2.355958	1.128928
1	-2.022286	2.119179	1.319981
1	-3.558714	2.328099	-0.679241
1	-2.893117	2.683482	-2.282666
1	-2.247279	4.968681	-1.498934
1	-3.972519	4.703077	-1.266234
1	-1.367930	4.373166	2.158014
1	-0.650750	4.769710	0.598082
1	-2.921489	5.761016	0.752284
1	-3.611162	4.184229	1.129994
46	3.287109	-0.854310	0.415333
17	5.223241	-1.256267	1.828353
17	4.150901	-2.160040	-1.422532
6	2.431750	-0.004560	1.931191
7	1.926678	0.470278	2.871585
6	1.331976	0.992706	4.038343
1	2.047265	1.642792	4.550381
1	1.048686	0.168371	4.699377
1	0.436998	1.563400	3.773568
7	1.559626	0.612578	-1.565649
6	1.742936	-0.365041	-0.782190
6	2.455404	2.630625	-0.469329
6	3.282260	3.890827	-0.686939
6	2.488806	1.739173	-1.711812
6	4.714806	3.543982	-1.075343
6	3.925162	1.368505	-2.072658
6	4.747357	2.633630	-2.297513
1	2.080691	2.325635	-2.551261
1	3.935236	0.720681	-2.958288
1	4.369587	0.780808	-1.254486
1	2.864934	2.059505	0.377592

1	1.415368	2.879242	-0.213202
1	2.820767	4.494011	-1.485805
1	3.263031	4.509840	0.219597
1	5.780184	2.360899	-2.548335
1	4.349369	3.178287	-3.169537
1	5.293598	4.458211	-1.261806
1	5.202769	3.030574	-0.231633
1	-0.112277	1.035419	-1.609482

INT9

Pd	-0.595858	-1.180713	-1.523449
Cl	-2.407738	-1.984961	-2.963639
Cl	0.727425	-0.307584	-3.326049
C	-1.524466	-1.962168	-0.028603
N	-2.055598	-2.503893	0.862207
C	-2.719755	-3.188669	1.900159
H	-3.754094	-3.385772	1.604412
H	-2.709525	-2.580613	2.809467
H	-2.212123	-4.138916	2.093797
N	1.051358	0.895575	-0.215372
N	2.164449	1.361572	0.413211
C	2.197396	2.683163	0.449832
H	3.026782	3.204377	0.909698
C	0.320179	1.990866	-0.603382
C	1.037096	3.150181	-0.187414
C	-0.909675	2.100363	-1.266891
C	0.530770	4.438913	-0.442599
C	-1.381340	3.377701	-1.497223
C	-0.675833	4.536109	-1.096359
H	1.084557	5.319970	-0.128081
H	-1.099665	5.514065	-1.311554
H	-2.332217	3.493479	-2.012587
H	-1.468509	1.226807	-1.593442
N	1.761184	-1.177629	0.332261
C	0.881692	-0.535079	-0.320378
C	3.082001	-3.138220	-0.269345
C	3.144128	-4.660075	-0.222648
C	1.773513	-2.629123	0.333194
C	2.950946	-5.183998	1.196231
C	1.599588	-3.140687	1.762639
C	1.653271	-4.662578	1.803772
H	0.940274	-3.018665	-0.282095
H	0.651078	-2.766424	2.175165
H	2.402681	-2.718217	2.387950
H	3.922346	-2.708715	0.299682
H	3.176073	-2.772572	-1.300949
H	2.355591	-5.072076	-0.871898
H	4.098763	-5.009637	-0.637506
H	1.538174	-5.017528	2.836780
H	0.798493	-5.068612	1.238168
H	2.964595	-6.281987	1.204993

H	3.797278	-4.856811	1.821834
H	2.796374	0.641432	0.768338

INT10

7	-2.085269	-1.549727	0.289035
6	-2.437212	-0.555828	-0.216453
46	-3.125073	1.073859	-0.989225
17	-4.048660	3.067229	-1.857444
17	-5.229752	0.287608	-0.306890
6	-2.337915	-2.548204	2.470744
6	-2.151309	-3.831383	3.271347
6	-1.863750	-2.754100	1.030801
6	-2.863100	-5.007153	2.612626
6	-2.585774	-3.922119	0.355599
6	-2.396619	-5.194253	1.174097
1	-0.778829	-2.935699	1.021220
1	-2.214347	-4.047045	-0.668740
1	-3.654437	-3.670534	0.285381
1	-3.401053	-2.266655	2.444206
1	-1.791841	-1.711908	2.923439
1	-1.075921	-4.054472	3.355180
1	-2.516766	-3.677576	4.294218
1	-2.937575	-6.018093	0.692438
1	-1.331441	-5.475244	1.167777
1	-2.696474	-5.926091	3.188935
1	-3.948872	-4.824453	2.621038
7	-0.263374	0.942234	-2.000029
7	-1.251392	1.765742	-1.554474
6	-0.754302	2.991568	-1.470101
1	-1.383802	3.798395	-1.118498
6	0.870202	1.651686	-2.260224
6	0.591923	2.994447	-1.903094
6	2.114297	1.251764	-2.760488
6	1.592113	3.973175	-2.037582
6	3.075568	2.238186	-2.885665
6	2.822155	3.581511	-2.527618
1	1.395362	5.007194	-1.763747
1	3.614894	4.317167	-2.642857
1	4.057162	1.971089	-3.272195
1	2.312936	0.219419	-3.037348
1	-0.499142	-0.002304	-2.275527

INT14

7	-2.746535	-1.278869	0.365078
6	-3.145538	-0.037128	0.385292
46	-4.783264	1.113738	0.399344
17	-6.312424	2.940920	0.476019
17	-6.509162	-0.453509	0.315397
6	-3.064541	-3.469026	-0.666900
6	-3.944460	-4.713596	-0.719803
6	-3.637272	-2.450206	0.310132
6	-4.140773	-5.316857	0.665755

6	-3.818478	-3.044677	1.703234
6	-4.702426	-4.285332	1.636933
1	-4.601799	-2.076253	-0.051493
1	-4.251208	-2.288965	2.371441
1	-2.829076	-3.309934	2.110763
1	-2.050349	-3.749903	-0.334563
1	-2.961590	-3.016590	-1.661634
1	-4.924609	-4.441815	-1.140386
1	-3.504683	-5.447499	-1.407095
1	-4.815578	-4.714457	2.640383
1	-5.710950	-3.986963	1.310738
1	-4.803626	-6.189921	0.609748
1	-3.172903	-5.683900	1.043519
7	-2.227069	0.999412	0.445398
7	-2.979580	2.168054	0.468694
6	-2.160572	3.185672	0.508367
1	-2.545347	4.197877	0.531538
6	-0.871309	1.304165	0.473517
6	-0.811057	2.719489	0.514085
6	0.280614	0.518894	0.466012
6	0.427944	3.369318	0.550111
6	1.495875	1.189573	0.503279
6	1.573571	2.591717	0.544661
1	0.479989	4.455113	0.581605
1	2.550171	3.068976	0.572604
1	2.414370	0.606700	0.500373
1	0.255344	-0.566592	0.433983
1	-1.752989	-1.499886	0.424441

INT15

7	-3.747206	-0.993102	0.969376
6	-2.770408	-1.339354	1.491636
46	-1.153199	-1.973091	2.442585
17	0.702238	-2.662152	3.668730
17	-2.618863	-2.845377	4.078789
6	-5.637888	-1.796510	-0.309949
6	-6.952616	-1.372186	-0.952554
6	-4.967413	-0.585426	0.342812
6	-7.874878	-0.695559	0.054809
6	-5.875308	0.087555	1.374156
6	-7.187744	0.494041	0.714998
1	-4.675868	0.140332	-0.430157
1	-5.362515	0.952777	1.810406
1	-6.063815	-0.626578	2.188777
1	-5.820524	-2.552076	0.467895
1	-4.959300	-2.242739	-1.046543
1	-6.743569	-0.679647	-1.782508
1	-7.436521	-2.250781	-1.396245
1	-7.840924	0.953407	1.466776
1	-6.989934	1.269858	-0.040982
1	-8.803028	-0.375020	-0.434578

1	-8.164260	-1.423647	0.828614
7	-0.478821	-0.174348	0.252034
7	0.020100	-1.191772	0.984474
6	1.265581	-1.575734	0.633392
1	1.784090	-2.367824	1.156847
6	0.510721	0.104237	-0.631921
6	1.637560	-0.758359	-0.440969
6	0.519421	1.088166	-1.644323
6	2.777704	-0.634919	-1.264458
6	1.645927	1.187835	-2.429549
6	2.766567	0.334437	-2.242811
1	3.640066	-1.285373	-1.127253
1	3.632592	0.454294	-2.891728
1	1.685520	1.940379	-3.215811
1	-0.337590	1.744025	-1.787603
1	-1.036598	-0.658018	3.179748

INT16

7	1.944507	1.936182	-0.295766
7	2.084568	0.596261	-0.431467
6	3.115510	0.096233	0.275375
6	3.734908	1.170382	0.931978
6	2.956779	2.303121	0.529833
6	4.836864	1.338837	1.795394
6	5.137699	2.611648	2.236905
6	4.365180	3.731777	1.835350
6	3.282797	3.595821	0.990470
46	0.734906	-0.459875	-1.540225
17	-0.927858	-1.634183	-2.794857
6	-0.654627	0.734581	-0.972344
7	-1.507857	1.452567	-0.620075
6	-2.531995	2.343821	-0.185623
6	-3.309390	2.885275	-1.386712
6	-4.405080	3.832908	-0.915286
6	-5.334300	3.159028	0.088079
6	-4.551229	2.598171	1.270046
6	-3.456190	1.643281	0.812238
17	2.438272	-1.978596	-2.237356
1	-2.017911	3.176108	0.318972
1	-2.869679	1.273664	1.662042
1	-3.898145	0.765188	0.318631
1	-3.749300	2.033950	-1.926755
1	-2.620035	3.384494	-2.078089
1	-3.943641	4.717843	-0.448919
1	-4.969245	4.199467	-1.782135
1	-5.219647	2.080407	1.969547
1	-4.095283	3.428376	1.832570
1	-6.097651	3.866859	0.436997
1	-5.873433	2.338801	-0.411850
1	3.349632	-0.960424	0.238942
1	5.438575	0.484750	2.106695

1	5.984551	2.765727	2.904646
1	4.637482	4.720071	2.205417
1	2.692367	4.457746	0.680195

INT16•IndH$^+$

6	-2.498045	-2.444335	3.526560
6	-1.134906	-2.635886	3.489321
6	-0.538273	-2.852772	2.231669
6	-1.345522	-2.889096	1.061202
6	-2.731399	-2.691816	1.100013
6	-3.280913	-2.467118	2.346260
6	0.782157	-3.038671	1.786149
7	0.743014	-3.189006	0.468368
7	-0.526850	-3.144932	0.006883
7	1.925521	1.918685	-0.279409
7	2.087120	0.584175	-0.419099
6	3.140760	0.097141	0.267592
6	3.756114	1.182960	0.906730
6	2.950531	2.301695	0.523454
6	4.876666	1.368459	1.743571
6	5.162303	2.645490	2.179098
6	4.359534	3.752022	1.798501
6	3.262574	3.600403	0.977506
46	0.733330	-0.460145	-1.507720
17	-0.947029	-1.656101	-2.765920
6	-0.640317	0.761023	-0.943486
7	-1.503857	1.448146	-0.562277
6	-2.539614	2.330075	-0.130669
6	-3.247567	2.939175	-1.341768
6	-4.353107	3.879096	-0.878214
6	-5.338977	3.172657	0.044808
6	-4.622640	2.540938	1.233375
6	-3.517479	1.592391	0.784920
17	2.361418	-2.152392	-2.046455
1	-2.039750	3.128128	0.438459
1	-2.976955	1.177094	1.644758
1	-3.943848	0.743764	0.228250
1	-3.669030	2.121784	-1.945068
1	-2.517181	3.462251	-1.970427
1	-3.902679	4.732845	-0.347916
1	-4.868120	4.295669	-1.752681
1	-5.331588	1.999915	1.872831
1	-4.183753	3.333826	1.859048
1	-6.105581	3.876226	0.394262
1	-5.867717	2.388322	-0.519487
1	3.403073	-0.952232	0.219806
1	5.502679	0.525778	2.036209
1	6.022307	2.815586	2.825406
1	4.624702	4.743952	2.162772
1	2.650259	4.451468	0.682100
1	1.509435	-3.210689	-0.221132

1	1.725518	-3.075161	2.314442
1	-0.531843	-2.616523	4.393453
1	-2.991233	-2.269802	4.479896
1	-4.353216	-2.300250	2.425715
1	-3.333314	-2.712678	0.195389
1	-0.685840	-2.930662	-0.991360

INT17

7	-2.750883	-1.220605	0.330103
6	-3.219203	-0.049769	0.438690
46	-4.842790	1.134974	0.541553
17	-6.390871	3.085839	0.649465
17	-6.541860	-0.506356	0.518941
6	-2.917135	-3.448514	-0.615481
6	-3.737616	-4.729933	-0.692145
6	-3.614864	-2.387279	0.232149
6	-4.049157	-5.268943	0.699239
6	-3.927633	-2.929854	1.626511
6	-4.745888	-4.213132	1.550678
1	-4.570470	-2.110381	-0.242019
1	-4.462549	-2.158581	2.196775
1	-2.977074	-3.120458	2.152126
1	-1.933298	-3.661495	-0.164863
1	-2.719838	-3.047028	-1.619127
1	-4.683062	-4.524104	-1.219296
1	-3.208617	-5.484401	-1.290668
1	-4.940505	-4.597877	2.561248
1	-5.730489	-3.985641	1.111553
1	-4.665171	-6.176483	0.631732
1	-3.107871	-5.567615	1.190323
7	-2.301572	1.034505	0.519531
7	-3.014791	2.203966	0.595506
6	-2.159973	3.206722	0.614983
1	-2.516940	4.227474	0.673436
6	-0.955351	1.286027	0.482215
6	-0.831243	2.700115	0.547716
6	0.151450	0.437565	0.398107
6	0.446796	3.279959	0.531188
6	1.397618	1.043061	0.382211
6	1.546290	2.444255	0.448190
1	0.564218	4.360885	0.580891
1	2.546634	2.871859	0.432019
1	2.287341	0.419381	0.316557
1	0.028066	-0.640510	0.346092

TS1

7	0.309222	-0.419738	1.545837
6	1.472048	-0.411515	1.921216
46	3.017003	-1.549370	2.443578
7	2.091623	1.340189	1.841111
17	4.827183	-3.051597	2.977098
17	3.668589	0.006544	4.148466

6	2.539326	-2.837517	1.081489
7	2.308546	-3.649273	0.274482
6	2.076029	-4.667157	-0.673595
6	-1.836495	-1.436962	2.129064
6	-2.736972	-2.648117	1.908283
6	-0.517206	-1.614347	1.386282
6	-2.966646	-2.913920	0.424444
6	-0.754832	-1.840530	-0.104410
6	-1.644080	-3.059398	-0.319955
1	1.512732	-5.479191	-0.204859
1	3.034892	-5.051260	-1.032788
1	1.505389	-4.263713	-1.515264
7	1.015374	2.250856	1.919033
1	0.011148	-2.491727	1.801396
1	0.208441	-1.950287	-0.621913
1	-1.235270	-0.941875	-0.520978
1	-2.326664	-0.525288	1.754557
1	-1.643385	-1.278424	3.197668
1	-2.271757	-3.533986	2.368971
1	-3.692288	-2.498107	2.427146
1	-1.815006	-3.212739	-1.393701
1	-1.119583	-3.958325	0.043914
1	-3.583482	-3.811826	0.287838
1	-3.534460	-2.076503	-0.010874
6	0.907953	2.776212	0.732249
1	2.670006	1.345426	2.698324
1	0.105825	3.481842	0.536625
6	2.737589	1.483610	0.579655
6	1.942957	2.351364	-0.176930
6	3.868800	0.858124	0.080686
6	2.279918	2.627926	-1.502576
6	4.199449	1.152632	-1.242243
6	3.417768	2.018370	-2.020705
1	1.677039	3.301694	-2.107404
1	3.712227	2.218461	-3.048699
1	5.088135	0.700506	-1.677476
1	4.466889	0.182021	0.690150

TS2

N	-1.316827	1.570504	0.446523
C	-0.409807	0.749886	0.048261
Pd	-0.238429	-1.174608	-0.352075
H	-0.208549	2.423282	0.411011
Cl	0.000652	-3.522435	-0.871937
Cl	-1.038534	-1.615848	1.851033
C	0.454940	-0.800999	-2.115407
N	0.892886	-0.603134	-3.179289
C	1.455769	-0.296928	-4.433658
H	0.803920	-0.666043	-5.230322
H	2.436694	-0.773194	-4.519442
H	1.563201	0.788928	-4.522895

C	-3.017333	1.557716	2.176638
C	-4.482909	1.278685	2.484274
C	-2.707795	1.245611	0.713552
C	-5.403774	2.055860	1.550984
C	-3.614530	2.039955	-0.224419
C	-5.080708	1.764156	0.089742
H	-2.843868	0.166319	0.538081
H	-3.381020	1.787790	-1.267181
H	-3.398478	3.112846	-0.098844
H	-2.788658	2.619380	2.364033
H	-2.357245	0.962565	2.820999
H	-4.672726	0.199214	2.374604
H	-4.696150	1.524785	3.532425
H	-5.720469	2.358535	-0.575691
H	-5.303340	0.707619	-0.127160
H	-6.454293	1.818314	1.764033
H	-5.284170	3.135191	1.739985
N	0.768281	1.703465	0.026045
N	1.264530	2.053035	-1.274522
C	2.558975	2.111661	-1.149080
H	3.155886	2.413238	-2.005802
C	1.878920	1.456284	0.887785
C	3.035570	1.753478	0.161891
C	1.890456	1.024622	2.202726
C	4.287310	1.642456	0.771090
C	3.147104	0.905660	2.793998
C	4.322600	1.214070	2.093207
H	5.201051	1.876467	0.229712
H	5.280902	1.109985	2.596646
H	3.216075	0.560988	3.823274
H	0.975272	0.776689	2.736673

TS3

7	-1.948070	0.307191	0.311977
6	-0.871412	-0.361494	0.247290
46	-0.565021	-2.337632	0.293346
1	-1.611058	2.006219	0.731706
8	-0.781439	2.562871	0.937419
1	-0.062901	1.677472	0.644790
17	-0.229748	-4.763603	0.284060
17	-1.101437	-2.243950	2.639439
6	-0.141136	-2.329847	-1.585886
7	0.124412	-2.347646	-2.724703
6	0.470725	-2.378970	-4.089818
1	1.219816	-3.157937	-4.258941
1	0.879104	-1.407063	-4.384444
1	-0.418259	-2.594923	-4.689372
1	-0.764473	2.680424	1.908745
6	-4.093052	0.264799	1.466883
6	-5.474006	-0.376431	1.522086
6	-3.236149	-0.378232	0.381148

6	-6.169008	-0.307976	0.167254
6	-3.925544	-0.300744	-0.980706
6	-5.309154	-0.938014	-0.922946
1	-3.074615	-1.440135	0.636518
1	-3.297336	-0.792545	-1.736933
1	-4.009341	0.757748	-1.275840
1	-4.191369	1.341431	1.246378
1	-3.583531	0.179717	2.436139
1	-5.368829	-1.429738	1.825970
1	-6.082153	0.109631	2.296142
1	-5.800040	-0.852969	-1.901365
1	-5.201088	-2.015499	-0.722345
1	-7.149308	-0.800624	0.213125
1	-6.361814	0.746899	-0.088269
7	0.347293	0.510955	0.088374
7	0.512516	0.929704	-1.284794
6	1.789715	0.852986	-1.527034
1	2.157962	1.172072	-2.498509
6	1.612192	0.070909	0.569987
6	2.556788	0.335361	-0.428455
6	1.946112	-0.489791	1.794468
6	3.908858	0.057704	-0.208552
6	3.294552	-0.776388	1.992739
6	4.261268	-0.499990	1.013064
1	4.656152	0.266450	-0.970928
1	5.303578	-0.737156	1.215028
1	3.602977	-1.232606	2.930802
1	1.191418	-0.727593	2.542091

TS4

46	2.223820	0.550390	-0.948419
1	-1.207336	-1.320104	-0.543872
17	4.476395	1.074435	-1.777971
17	1.110622	1.769424	-2.681689
6	3.077169	-0.403015	0.495651
7	3.635331	-0.959801	1.361466
6	4.376039	-1.601354	2.374387
1	3.757984	-1.714066	3.270055
1	5.258619	-1.001529	2.615718
1	4.695895	-2.588495	2.026485
7	-0.029336	-1.333937	-0.456585
7	0.472101	-1.874978	-1.703146
6	1.081998	-2.978117	-1.397852
1	1.523343	-3.569603	-2.195137
6	0.389199	-2.217962	0.601480
6	1.093220	-3.270231	0.014933
6	0.176999	-2.107806	1.963631
6	1.627158	-4.282645	0.812400
6	0.714538	-3.126181	2.751389
6	1.429368	-4.191809	2.186460
1	2.177337	-5.112069	0.374046

1	1.833735	-4.962790	2.838874
1	0.576782	-3.089464	3.830000
1	-0.373827	-1.274588	2.394870
7	-3.192166	-0.179672	0.086036
7	-2.643782	-1.233513	-0.559616
6	-3.625394	-1.939111	-1.100534
1	-2.537968	0.492643	0.487167
1	-3.399851	-2.835706	-1.664530
6	-4.543567	-0.177796	-0.049105
6	-4.869336	-1.328354	-0.815446
6	-5.522430	0.707072	0.423491
6	-6.215350	-1.609239	-1.112850
6	-6.833792	0.404141	0.110323
6	-7.179779	-0.739598	-0.646156
1	-6.483021	-2.486706	-1.697683
1	-8.228620	-0.930462	-0.862958
1	-7.625637	1.066207	0.455519
1	-5.260625	1.588265	1.004371
7	-0.448539	0.773951	0.416158
6	0.405996	0.114390	-0.238370
6	-1.334830	3.046070	0.460966
6	-1.060159	4.487621	0.869411
6	-0.137952	2.156958	0.782069
6	-0.700002	4.579443	2.348147
6	0.208272	2.228856	2.268381
6	0.475748	3.669914	2.689148
1	0.733130	2.511904	0.203229
1	1.080421	1.590455	2.473768
1	-0.633603	1.815869	2.849250
1	-2.213413	2.670368	1.014765
1	-1.575420	2.974333	-0.608064
1	-0.227959	4.878827	0.263597
1	-1.930668	5.116409	0.641728
1	0.699380	3.712208	3.763415
1	1.376570	4.031918	2.169750
1	-0.470421	5.617239	2.623499
1	-1.573772	4.283580	2.951730

***E*-TS5**

46	-0.234088	-1.438099	-0.333993
1	1.062316	1.142719	-0.175460
17	1.222131	-3.358250	-0.907440
17	-1.271217	-1.362046	-2.485029
6	0.693555	-1.353736	1.361095
7	1.361884	-1.267044	2.318556
6	2.212319	-1.225200	3.442581
1	3.156620	-1.722773	3.200851
1	1.736788	-1.738357	4.283472
1	2.407875	-0.184568	3.718613
7	-2.557879	0.308082	0.061470
7	-3.309599	1.285412	-0.574001

6	-4.558383	0.885695	-0.501089
1	-5.335122	1.499269	-0.945921
6	-3.372015	-0.722412	0.513405
6	-4.692536	-0.360847	0.173765
6	-3.092140	-1.897646	1.216600
6	-5.766723	-1.188352	0.528099
6	-4.169989	-2.704994	1.550388
6	-5.492062	-2.358964	1.213423
1	-6.787341	-0.913136	0.269214
1	-6.305596	-3.022219	1.500604
1	-3.984303	-3.631498	2.090647
1	-2.076854	-2.176432	1.489506
7	-0.430734	1.377911	-0.010603
6	-1.158377	0.321409	-0.006556
6	0.005858	3.548946	1.038669
6	-0.497655	4.980620	1.188123
6	-0.921355	2.754951	0.122780
6	-0.623979	5.666081	-0.168807
6	-0.998623	3.420184	-1.250348
6	-1.495636	4.854941	-1.121306
1	-1.927196	2.749464	0.566312
1	-1.652493	2.830192	-1.903005
1	0.007636	3.407326	-1.701364
1	1.016613	3.566581	0.598333
1	0.088191	3.053838	2.017039
1	-1.482212	4.965357	1.681752
1	0.170059	5.550019	1.848346
1	-1.533826	5.331377	-2.109871
1	-2.531231	4.837918	-0.745016
1	-1.026849	6.680506	-0.047820
1	0.379959	5.781567	-0.608517
1	2.051071	-0.804689	-1.310756
7	2.689420	-0.135261	-0.875624
7	2.186719	0.918282	-0.193119
6	3.159726	1.499553	0.497488
1	2.949658	2.377364	1.095494
6	4.009695	-0.274046	-0.590618
6	4.359058	0.792918	0.280333
6	4.939862	-1.230079	-1.019381
6	5.688844	0.924381	0.726232
6	6.230152	-1.077977	-0.554125
6	6.604686	-0.013539	0.302157
1	5.976493	1.742395	1.382760
1	7.639532	0.057021	0.629387
1	6.987079	-1.798427	-0.856867
1	4.651258	-2.046241	-1.676285

***Z*-TS5**

Pd	-2.521855	-0.554603	-0.081043
H	1.836952	-0.261141	-0.387202
Cl	-4.840458	-1.342111	0.135273

Cl	-2.342796	-1.176798	-2.394216
C	-2.597352	-0.036005	1.769653
N	-2.664551	0.273975	2.896301
C	-2.809821	0.620710	4.254887
H	-3.777986	0.265950	4.619582
H	-2.756785	1.707662	4.364937
H	-2.009587	0.157761	4.839559
N	-0.367269	1.460123	-0.147713
N	0.831654	1.951463	0.333786
C	0.739233	3.263601	0.286151
H	1.571481	3.868896	0.632122
C	-1.212941	2.499219	-0.505101
C	-0.511415	3.694753	-0.229446
C	-2.491255	2.519758	-1.075617
C	-1.099357	4.941958	-0.488314
C	-3.051394	3.763935	-1.319769
C	-2.371654	4.962951	-1.028230
H	-0.560454	5.863080	-0.274038
H	-2.855414	5.913905	-1.242278
H	-4.045292	3.810744	-1.760318
H	-3.025161	1.607342	-1.333022
N	0.423359	-0.695448	-0.207516
C	-0.603494	0.073750	-0.185045
C	0.941727	-2.848050	-1.268106
C	0.792435	-4.359548	-1.144248
C	0.261747	-2.144341	-0.097737
C	1.344195	-4.862435	0.185271
C	0.828265	-2.630081	1.236170
C	0.691019	-4.143485	1.360529
H	-0.813573	-2.391457	-0.126965
H	0.309305	-2.120003	2.060814
H	1.891219	-2.340643	1.301774
H	2.014036	-2.587512	-1.273416
H	0.513056	-2.482965	-2.210531
H	-0.274951	-4.619924	-1.221233
H	1.292981	-4.857868	-1.984906
H	1.120932	-4.485086	2.311559
H	-0.379030	-4.402387	1.392752
H	1.201507	-5.947507	0.274775
H	2.432594	-4.687871	0.214092
H	2.741075	1.294867	1.117631
N	3.432042	0.736299	0.618427
N	2.960086	-0.009443	-0.404059
C	3.970153	-0.450628	-1.137237
H	3.784288	-1.097911	-1.984889
C	4.783493	0.843400	0.515873
C	5.172606	0.063241	-0.606520
C	5.712653	1.540172	1.298833
C	6.533611	-0.038192	-0.951017
C	7.038255	1.424658	0.929434

C	7.447679	0.645089	-0.177892
H	6.845684	-0.636725	-1.803521
H	8.506671	0.589574	-0.419172
H	7.794659	1.949700	1.509196
H	5.404896	2.137353	2.152968

TS6

46	3.289607	-2.433048	1.011825
1	-0.452522	0.249104	0.545314
17	5.564671	-3.181134	1.176790
17	2.769324	-3.052106	3.245644
6	3.688825	-1.929326	-0.819923
7	3.945303	-1.612326	-1.913551
6	4.298107	-1.251760	-3.230756
1	3.600110	-1.714207	-3.934103
1	5.313512	-1.598694	-3.440724
1	4.253241	-0.163986	-3.334670
7	0.398366	0.801439	0.508728
7	1.573011	0.156189	0.617004
6	2.544493	1.052816	0.599804
1	3.576674	0.730025	0.683240
6	0.586816	2.147803	0.421301
6	1.991527	2.352550	0.473787
6	-0.319400	3.208743	0.297071
6	2.507625	3.658419	0.396234
6	0.220899	4.479428	0.225949
6	1.615106	4.704808	0.273800
1	3.580623	3.834514	0.434444
1	1.986229	5.725723	0.213949
1	-0.447904	5.332727	0.132022
1	-1.393012	3.040223	0.261997
7	0.228714	-2.049365	0.825827
6	1.422775	-1.884008	0.859885
6	-1.479895	-3.130409	2.186996
6	-2.387209	-4.349166	2.311298
6	-0.592668	-3.258100	0.953742
6	-3.221437	-4.553233	1.051875
6	-1.424566	-3.432895	-0.312325
6	-2.335304	-4.648805	-0.184669
1	0.076473	-4.124145	1.075119
1	-0.763446	-3.525467	-1.183657
1	-2.028107	-2.523769	-0.462876
1	-2.086975	-2.216532	2.088614
1	-0.857702	-3.007539	3.082162
1	-1.769875	-5.243205	2.490170
1	-3.032218	-4.239686	3.192213
1	-2.943086	-4.751164	-1.092859
1	-1.717651	-5.558371	-0.121706
1	-3.842973	-5.453203	1.146265
1	-3.915610	-3.705473	0.936063

TS7

46	0.692094	-3.078788	1.567998
1	-2.172242	-0.309416	1.075296
17	2.368062	-4.816234	1.767818
17	0.737904	-2.534501	3.889094
6	0.553884	-3.530422	-0.301777
7	0.429595	-3.828884	-1.424754
6	0.301444	-4.221517	-2.773240
1	0.203714	-3.334151	-3.405314
1	-0.587251	-4.849846	-2.887706
1	1.187494	-4.788084	-3.072702
7	-1.366663	0.574532	1.396641
7	-0.334133	-0.281173	1.613737
6	0.778445	0.342696	2.021571
1	1.677016	-0.219143	2.242919
6	-0.903266	1.808943	1.679168
6	0.468619	1.706400	2.084440
6	-1.557019	3.051615	1.613605
6	1.189715	2.866787	2.440361
6	-0.820313	4.161269	1.964603
6	0.536099	4.074973	2.375657
1	2.227993	2.797769	2.756460
1	1.061716	4.988449	2.644592
1	-1.292957	5.140924	1.927779
1	-2.595777	3.123706	1.302260
7	-1.912255	-1.674982	0.993974
6	-0.681031	-1.652785	1.354218
6	-3.783220	-3.113334	1.616858
6	-4.585226	-4.347984	1.222824
6	-2.638536	-2.878104	0.633001
6	-5.094131	-4.251940	-0.211063
6	-3.156407	-2.759257	-0.798962
6	-3.949932	-4.000772	-1.186820
1	-1.941446	-3.732263	0.693778
1	-2.313549	-2.595605	-1.485283
1	-3.797876	-1.866239	-0.869484
1	-4.434761	-2.224979	1.617930
1	-3.377163	-3.212591	2.632104
1	-3.947027	-5.239838	1.323718
1	-5.420189	-4.486696	1.921557
1	-4.328524	-3.897568	-2.212065
1	-3.276152	-4.873291	-1.190044
1	-5.637022	-5.165728	-0.485817
1	-5.818615	-3.425136	-0.284860

TS8

46	-2.649500	-0.507470	-0.485823
1	1.683205	1.675473	0.021071
17	-4.821236	-1.378745	-1.232631
17	-2.166858	0.587220	-2.561994
6	-2.926840	-1.393707	1.198582
7	-3.075391	-1.982340	2.199766

6	-3.305317	-2.707595	3.386086
1	-4.361292	-2.986357	3.444399
1	-3.042424	-2.092570	4.251770
1	-2.691934	-3.614264	3.386441
7	0.460052	2.219785	0.349657
7	-0.738288	1.570245	0.299289
6	-1.779147	2.418036	0.428988
1	-2.795376	2.043635	0.402176
6	0.158317	3.524833	0.510449
6	-1.257653	3.700827	0.579578
6	1.033288	4.626059	0.606295
6	-1.809626	4.988091	0.759049
6	0.463795	5.868035	0.776661
6	-0.943037	6.051025	0.855229
1	-2.886996	5.127580	0.813654
1	-1.334826	7.056800	0.990228
1	1.109074	6.741169	0.854056
1	2.111957	4.493113	0.551666
7	0.233055	-0.517936	0.321816
6	-0.831950	0.147447	0.124074
6	1.081726	-2.338612	-1.082178
6	1.106619	-3.850643	-1.275013
6	0.212914	-1.962034	0.117418
6	1.559057	-4.570493	-0.009218
6	0.681393	-2.672502	1.385442
6	0.694889	-4.182756	1.185886
1	-0.815958	-2.300800	-0.104069
1	0.035119	-2.388197	2.228545
1	1.698960	-2.329823	1.645357
1	2.106304	-1.963966	-0.923289
1	0.696866	-1.832731	-1.977812
1	0.096087	-4.194781	-1.546162
1	1.756911	-4.109442	-2.120717
1	1.045632	-4.682410	2.098722
1	-0.337716	-4.530187	1.018096
1	1.539908	-5.658004	-0.158936
1	2.608316	-4.305014	0.204127
1	2.267579	-0.368632	0.969633
7	3.075603	0.126002	0.587317
7	2.781697	1.219601	-0.148310
6	3.850028	1.584186	-0.838774
1	3.820204	2.467185	-1.464192
6	4.344793	-0.281990	0.319344
6	4.892137	0.663062	-0.589860
6	5.075511	-1.384043	0.781911
6	6.218109	0.515733	-1.036849
6	6.370567	-1.503951	0.315939
6	6.938888	-0.566709	-0.578377
1	6.653072	1.233769	-1.728145
1	7.964996	-0.710607	-0.909152

1	6.973769	-2.347489	0.645545
1	4.645269	-2.105171	1.472735

***Z*-TS9**

46	-2.850829	0.053685	-0.089141
1	1.434577	-0.727781	-0.401541
17	-5.282654	-0.140708	0.078429
17	-2.866217	0.457448	-2.448201
6	-2.761128	-0.271357	1.807782
7	-2.726496	-0.491384	2.956390
6	-2.733621	-0.764557	4.339211
1	-3.640420	-1.319524	4.596431
1	-2.712729	0.175294	4.898618
1	-1.854497	-1.361684	4.599531
7	1.019371	1.765318	0.029737
7	-0.312139	1.518529	-0.157792
6	-1.048410	2.647662	-0.341177
1	-2.113180	2.586628	-0.529843
6	1.111666	3.105987	-0.046089
6	-0.165010	3.715188	-0.280052
6	2.274980	3.902576	0.072507
6	-0.281639	5.120117	-0.396253
6	2.128462	5.263942	-0.046292
6	0.861435	5.871511	-0.276649
1	-1.248766	5.584794	-0.576996
1	0.807951	6.954796	-0.362438
1	3.006206	5.903217	0.031653
1	3.250829	3.447606	0.236904
7	-0.003156	-0.769416	-0.273454
6	-0.837173	0.200230	-0.192992
6	-0.152970	-2.780717	-1.656853
6	-0.563581	-4.247520	-1.695851
6	-0.487945	-2.152509	-0.304878
6	0.059673	-5.031508	-0.547219
6	0.122258	-2.942720	0.852321
6	-0.296716	-4.407003	0.796848
1	-1.585560	-2.152179	-0.189438
1	-0.174417	-2.482743	1.805853
1	1.220955	-2.873816	0.800914
1	0.931430	-2.695321	-1.836160
1	-0.646034	-2.211820	-2.455181
1	-1.660449	-4.315444	-1.629458
1	-0.285972	-4.686132	-2.662949
1	0.172088	-4.960893	1.620725
1	-1.384664	-4.479210	0.952189
1	-0.260687	-6.081051	-0.579363
1	1.156639	-5.037604	-0.663678
1	2.690169	0.901864	0.681391
7	3.254691	0.096018	0.401904
7	2.617374	-0.799738	-0.379572
6	3.492429	-1.689120	-0.830244

1	3.166906	-2.513916	-1.452140
6	4.583562	-0.174673	0.427573
6	4.776397	-1.345386	-0.355873
6	5.644990	0.482857	1.064167
6	6.069320	-1.883714	-0.500576
6	6.899598	-0.068814	0.897230
6	7.112704	-1.237601	0.127788
1	6.234608	-2.781404	-1.092022
1	8.123421	-1.628257	0.034376
1	7.753162	0.409313	1.373526
1	5.484179	1.378683	1.658645

TS10

46	-1.034188	1.606970	-0.013840
1	0.322314	-2.015393	0.378004
17	-2.469733	3.510527	-0.452463
17	-0.498891	1.285103	-2.326610
6	-1.474678	1.828598	1.843954
7	-1.763612	1.982319	2.966749
6	-2.170631	2.234737	4.293185
1	-2.985085	2.964562	4.287504
1	-1.329079	2.633582	4.866784
1	-2.517825	1.305521	4.754320
7	1.706597	0.376562	0.110408
7	1.256491	0.128010	1.411312
6	2.024856	-0.812379	2.002281
1	1.790287	-1.165994	2.999060
6	2.774597	-0.456687	-0.086525
6	3.067604	-1.149552	1.127513
6	3.561351	-0.603763	-1.238190
6	4.201012	-1.990965	1.200229
6	4.644932	-1.450332	-1.142182
6	4.961832	-2.132036	0.062625
1	4.453320	-2.512369	2.120484
1	5.834519	-2.781497	0.079256
1	5.282448	-1.601163	-2.010723
1	3.318751	-0.068349	-2.152557
7	-0.277244	-1.228284	0.131017
6	0.142717	0.013931	0.371755
6	-1.425227	-2.690714	-1.462113
6	-2.778074	-3.078673	-2.048577
6	-1.586191	-1.580905	-0.430094
6	-3.767334	-3.475891	-0.959096
6	-2.565947	-1.979523	0.670773
6	-3.915257	-2.368066	0.077522
1	-1.956101	-0.677430	-0.933664
1	-2.675076	-1.148440	1.382297
1	-2.144072	-2.829655	1.232624
1	-0.968897	-3.569023	-0.974209
1	-0.733019	-2.364268	-2.249264
1	-3.180663	-2.223047	-2.612872

1	-2.649472	-3.893348	-2.772818
1	-4.603056	-2.672292	0.877276
1	-4.364236	-1.482179	-0.398173
1	-4.742915	-3.719889	-1.399178
1	-3.411522	-4.393230	-0.462282

TS11

N	-0.566861	-0.929349	-1.434155
N	0.665973	-1.208835	-0.863385
C	0.844427	-2.533775	-0.649840
H	1.783760	-2.905081	-0.256420
C	-1.164194	-2.149108	-1.551047
C	-0.309214	-3.184912	-1.072398
C	-2.449718	-2.433168	-2.035517
C	-0.727473	-4.533298	-1.099492
C	-2.837967	-3.757501	-2.037379
C	-1.989033	-4.797958	-1.577313
H	-0.069906	-5.326136	-0.752081
H	-2.349801	-5.823015	-1.611218
H	-3.826049	-4.019565	-2.411926
H	-3.098601	-1.641630	-2.405587
Pd	-2.355174	-0.443130	1.090795
Cl	-3.248053	-1.286797	3.163384
Cl	-0.232047	0.084825	2.045133
C	-4.084811	-0.817896	0.299338
N	-5.142716	-1.034250	-0.145709
C	-6.415373	-1.317624	-0.680771
H	-7.066658	-1.699058	0.110436
H	-6.319018	-2.071145	-1.468998
H	-6.846724	-0.403888	-1.100625
N	-1.674200	1.368688	-1.278755
C	-1.680710	0.371606	-0.576177
C	-3.761707	2.426223	-1.918505
C	-4.593963	3.701729	-1.863712
C	-2.459047	2.599034	-1.136836
C	-4.859774	4.131901	-0.425836
C	-2.731809	3.005250	0.308183
C	-3.556395	4.286633	0.348700
H	-1.868119	3.389716	-1.624745
H	-1.785589	3.125974	0.851967
H	-3.278768	2.190779	0.811078
H	-4.320923	1.590644	-1.465991
H	-3.539650	2.140431	-2.954678
H	-4.056430	4.504885	-2.391685
H	-5.535800	3.551589	-2.407168
H	-3.754139	4.565435	1.391238
H	-2.970458	5.111677	-0.087222
H	-5.431264	5.068800	-0.407019
H	-5.486348	3.373133	0.070188
Pd	3.448530	-0.764770	-0.023871
Cl	5.633343	-1.428838	0.714901

Cl	3.795851	-1.547837	-2.261876
C	3.111842	-0.204338	1.797118
N	2.957949	0.114478	2.908182
C	2.674787	0.507589	4.231282
H	3.609200	0.626962	4.786519
H	2.055520	-0.256613	4.709836
H	2.128521	1.455735	4.215187
N	1.324614	0.929707	-1.243357
C	1.654301	-0.221769	-0.731015
C	1.841527	2.811815	0.256524
C	2.455848	4.203464	0.335494
C	2.033266	2.223225	-1.139289
C	3.922095	4.186562	-0.079841
C	3.500736	2.180733	-1.546008
C	4.089506	3.586519	-1.470833
H	1.499179	2.859903	-1.859416
H	3.603464	1.753386	-2.550987
H	4.058319	1.520334	-0.863437
H	2.322749	2.143497	0.986550
H	0.772995	2.830147	0.509025
H	1.897213	4.883231	-0.327706
H	2.340704	4.600389	1.352146
H	5.148480	3.552391	-1.754623
H	3.589519	4.232576	-2.210688
H	4.341282	5.200556	-0.047447
H	4.497781	3.586496	0.643016
H	0.405719	1.000611	-1.691229

TS12

N	0.758573	-0.806620	-1.117440
N	-0.578649	-1.064032	-0.690064
C	-0.824519	-2.382476	-0.803959
H	-1.808060	-2.763359	-0.553518
C	1.272308	-2.027821	-1.502860
C	0.293419	-3.032589	-1.309096
C	2.537690	-2.334838	-2.016992
C	0.563696	-4.382587	-1.618294
C	2.779524	-3.666232	-2.304997
C	1.813666	-4.682010	-2.104603
H	-0.197106	-5.144835	-1.472865
H	2.068607	-5.708955	-2.353212
H	3.750746	-3.939218	-2.711752
H	3.284686	-1.566550	-2.202015
Pd	3.352970	-0.357809	0.217569
Cl	5.401576	-1.110434	1.272456
Cl	4.510116	0.721752	-1.600653
C	2.368966	-1.297576	1.595456
N	1.757577	-1.895775	2.390922
C	1.050268	-2.616975	3.375809
H	1.721588	-3.344321	3.841026
H	0.205972	-3.133526	2.910270

H	0.671657	-1.924505	4.132466
N	1.071593	1.420618	-0.616081
C	1.605666	0.265253	-0.567307
C	2.601131	2.620999	1.034725
C	2.911283	4.029766	1.532046
C	1.568619	2.688093	-0.082644
C	3.410668	4.911068	0.393304
C	2.052098	3.560704	-1.244588
C	2.401091	4.958682	-0.747370
H	0.663464	3.167975	0.332544
H	1.280206	3.603912	-2.025427
H	2.934323	3.076985	-1.688862
H	3.529569	2.168475	0.658687
H	2.233347	1.983166	1.850915
H	2.003171	4.474037	1.971865
H	3.654623	3.978834	2.337366
H	2.785048	5.560983	-1.580899
H	1.482397	5.458817	-0.399069
H	3.623253	5.925105	0.756843
H	4.363351	4.505965	0.016894
Pd	-3.206559	-0.856465	0.546300
Cl	-5.039093	-1.645968	1.889127
Cl	-1.726800	-0.543805	2.408356
C	-4.312802	-1.205393	-1.006073
N	-5.009184	-1.416787	-1.919619
C	-5.915019	-1.641250	-2.978088
H	-6.413167	-2.603853	-2.832763
H	-5.373994	-1.647313	-3.928280
H	-6.662357	-0.842390	-2.985841
N	-1.399072	1.087774	-0.841585
C	-1.638865	-0.117378	-0.471594
C	-3.624868	2.062162	-1.466929
C	-4.415742	3.365186	-1.433667
C	-2.309624	2.240208	-0.717279
C	-4.670184	3.812207	0.001530
C	-2.563237	2.638882	0.735789
C	-3.366475	3.934403	0.781374
H	-1.754408	3.056814	-1.204914
H	-1.612242	2.738334	1.276282
H	-3.119641	1.828562	1.233107
H	-4.213902	1.276700	-0.973180
H	-3.434068	1.732235	-2.497595
H	-3.854512	4.147132	-1.969456
H	-5.362833	3.237266	-1.973325
H	-3.563961	4.207007	1.825452
H	-2.760934	4.750786	0.355424
H	-5.213385	4.765909	0.014449
H	-5.319913	3.074840	0.499036
H	-0.237223	1.365195	-0.946187

TS13

7	-0.673086	-1.238164	-0.808107
7	0.455695	-1.262935	-1.645061
6	0.629348	-2.509949	-2.048939
1	1.486155	-2.754839	-2.663363
6	-1.176035	-2.543153	-0.696813
6	-0.360674	-3.361182	-1.510332
6	-2.245219	-3.075059	0.020531
6	-0.600328	-4.738179	-1.623016
6	-2.462819	-4.441778	-0.102634
6	-1.660388	-5.266369	-0.912139
1	0.043854	-5.360130	-2.239485
1	-1.872709	-6.331503	-0.962016
1	-3.276169	-4.888632	0.464943
1	-2.868945	-2.469774	0.673976
46	-2.900256	0.043236	0.646254
17	-4.828861	0.261190	2.067573
17	-1.369652	-0.031715	2.473241
6	-4.110544	-0.069850	-0.863983
7	-4.830443	-0.160218	-1.778908
6	-5.725020	-0.227486	-2.867549
1	-6.751082	-0.147625	-2.498651
1	-5.596583	-1.180722	-3.387989
1	-5.517554	0.596308	-3.556799
7	-0.813386	0.999066	-1.204048
6	-1.311951	-0.040020	-0.569982
6	-2.560436	2.828953	-1.407846
6	-2.708871	4.346157	-1.320154
6	-1.134123	2.437056	-1.038175
6	-2.342417	4.857697	0.069085
6	-0.765632	2.922970	0.357850
6	-0.931803	4.433503	0.458379
1	-0.460439	2.918567	-1.762175
1	0.263527	2.616072	0.585210
1	-1.414967	2.416941	1.088863
1	-3.264356	2.364237	-0.703178
1	-2.811276	2.458932	-2.411956
1	-2.057841	4.822078	-2.070951
1	-3.737390	4.628875	-1.577376
1	-0.695892	4.766003	1.477753
1	-0.202647	4.926153	-0.206338
1	-2.439775	5.950023	0.110810
1	-3.056205	4.450906	0.802917
46	2.616762	-1.172611	0.569175
17	3.609262	-2.015373	2.585425
17	3.399838	-3.040883	-0.697856
6	2.037938	0.338995	1.636395
7	1.771332	1.236469	2.332708
6	1.348749	2.254552	3.210112
1	1.713222	3.223706	2.853890
1	1.749925	2.059711	4.208803

1	0.253692	2.254114	3.248672
7	2.173519	0.549957	-1.925895
6	1.965320	-0.337246	-1.107063
6	2.739029	2.805444	-1.204693
6	3.801087	3.898235	-1.215681
6	3.255100	1.543128	-1.896674
6	5.103507	3.411930	-0.590990
6	4.549284	1.051845	-1.256087
6	5.604539	2.150175	-1.284958
1	3.454086	1.792237	-2.949931
1	4.900130	0.146033	-1.767780
1	4.345315	0.764267	-0.212479
1	2.477861	2.548082	-0.164904
1	1.815466	3.148898	-1.692369
1	3.988703	4.208020	-2.255708
1	3.422173	4.785455	-0.691154
1	6.527650	1.788296	-0.815121
1	5.860016	2.385687	-2.330697
1	5.864275	4.202253	-0.630829
1	4.933639	3.193325	0.476037
1	-0.026378	0.833104	-1.835622

TS14

Pd	-0.224151	-1.523940	-0.131986
Cl	-1.298886	-3.666968	0.007996
Cl	1.083076	-2.137689	-2.019530
C	-1.283151	-0.997893	1.410729
N	-1.959960	-0.665597	2.301516
C	-2.844578	-0.254353	3.319281
H	-3.344767	-1.129743	3.742614
H	-3.591290	0.422802	2.891790
H	-2.286666	0.262955	4.104692
N	-0.769317	1.579161	-0.529236
N	-0.386351	2.783466	-0.075315
C	-1.398587	3.590430	0.264138
H	-1.220683	4.595610	0.623360
C	-2.122784	1.592983	-0.454679
C	-2.572993	2.859161	0.036894
C	-3.034843	0.575275	-0.798106
C	-3.953790	3.107627	0.192646
C	-4.374041	0.845916	-0.623139
C	-4.831030	2.097893	-0.132724
H	-4.309655	4.070367	0.554308
H	-5.901523	2.259004	-0.023294
H	-5.107358	0.082199	-0.874534
H	-2.686097	-0.381600	-1.184876
N	1.696053	0.882369	-0.152704
C	0.709192	0.188363	-0.210607
C	3.925471	1.028580	-1.126957
C	5.403675	0.723396	-0.911132
C	3.104663	0.522652	0.053266

C	5.908251	1.309828	0.402493
C	3.588595	1.128776	1.366159
C	5.067786	0.824586	1.577923
H	3.180541	-0.574527	0.105095
H	2.984132	0.745942	2.198832
H	3.431034	2.218567	1.327399
H	3.772707	2.115492	-1.219052
H	3.560033	0.572997	-2.055418
H	5.549134	-0.368129	-0.903458
H	5.986680	1.106000	-1.758462
H	5.407722	1.281947	2.515928
H	5.199951	-0.262060	1.697209
H	6.964217	1.052347	0.556495
H	5.859196	2.409477	0.351979
H	0.614716	2.963263	-0.031413

TS15

Pd	-0.776487	-1.044553	0.459511
Cl	-2.833725	-2.220047	1.005112
Cl	-0.863447	-1.607505	-1.856540
C	-0.673322	-0.532800	2.317721
N	-0.643766	-0.225911	3.445649
C	-0.673435	0.098688	4.817936
H	-1.497060	-0.437083	5.297908
H	-0.820453	1.176040	4.936587
H	0.272179	-0.191822	5.284540
N	0.885155	1.341825	-0.109198
N	2.113502	1.864692	-0.389122
C	2.015305	3.171612	-0.525455
H	2.888895	3.771120	-0.749787
C	-0.044452	2.346450	-0.071948
C	0.664070	3.552008	-0.329327
C	-1.425054	2.326436	0.148948
C	-0.022820	4.777712	-0.356487
C	-2.070831	3.549886	0.112702
C	-1.384571	4.759704	-0.132261
H	0.507451	5.706352	-0.554055
H	-1.946424	5.690630	-0.149638
H	-3.146609	3.576939	0.272090
H	-1.961950	1.396294	0.326563
N	2.112670	-0.485019	-0.145963
C	0.910004	-0.075686	0.033663
C	3.350764	-2.258066	-1.270890
C	3.821888	-3.704427	-1.178923
C	2.502290	-1.883617	-0.059403
C	4.578288	-3.961248	0.119163
C	3.260160	-2.133512	1.243666
C	3.734476	-3.579633	1.330168
H	1.584539	-2.497250	-0.060579
H	2.614048	-1.878185	2.095822
H	4.125450	-1.451759	1.284354

H	4.221371	-1.582736	-1.310089
H	2.774125	-2.088329	-2.189270
H	2.947164	-4.371830	-1.229906
H	4.447033	-3.949700	-2.047364
H	4.297643	-3.733134	2.260086
H	2.857616	-4.243851	1.384911
H	4.884263	-5.013787	0.182889
H	5.506098	-3.366376	0.121785
H	2.705559	0.807623	-0.392912

TS16

Pd	-2.279532	-0.858273	-0.503757
H	1.410735	2.114108	-0.024352
Cl	-4.201960	-2.180119	-1.301905
Cl	-2.107297	0.455027	-2.503361
C	-2.278307	-1.943201	1.086207
N	-2.238317	-2.643590	2.023802
C	-2.216403	-3.509832	3.135325
H	-3.196470	-3.980800	3.253458
H	-1.973651	-2.942989	4.039185
H	-1.459495	-4.285411	2.981149
N	-0.885085	1.578355	0.371917
N	0.166913	2.452187	0.268225
C	-0.286513	3.682474	0.409552
H	0.399708	4.519179	0.342979
C	-2.043603	2.296952	0.582900
C	-1.681206	3.666406	0.621118
C	-3.373571	1.894974	0.763944
C	-2.653865	4.659615	0.826592
C	-4.311795	2.892822	0.958726
C	-3.963557	4.260141	0.990777
H	-2.373290	5.710300	0.851206
H	-4.742498	5.003260	1.146009
H	-5.354279	2.609325	1.089304
H	-3.666381	0.847472	0.737759
N	0.509364	-0.237695	0.374800
C	-0.677749	0.180454	0.176572
C	1.781491	-1.628703	-1.178320
C	2.203496	-3.051629	-1.522349
C	0.856839	-1.612986	0.040895
C	2.850973	-3.743518	-0.327762
C	1.510308	-2.303617	1.236489
C	1.925235	-3.727050	0.884086
H	-0.051003	-2.184134	-0.229092
H	0.821108	-2.289510	2.093370
H	2.403590	-1.733877	1.546284
H	2.672801	-1.017078	-0.963675
H	1.269339	-1.152738	-2.025479
H	1.317156	-3.624834	-1.836201
H	2.886364	-3.043325	-2.381965
H	2.405165	-4.207003	1.747531

H	1.023147	-4.319726	0.660058
H	3.129153	-4.774304	-0.583885
H	3.788390	-3.221636	-0.071493
H	2.441320	0.392425	1.193830
N	3.131952	0.998176	0.748547
N	2.629211	1.864745	-0.160335
C	3.615587	2.317145	-0.916568
H	3.420903	3.057640	-1.681736
C	4.463327	0.817603	0.530624
C	4.822813	1.686583	-0.533789
C	5.393695	-0.017531	1.162047
C	6.159623	1.739826	-0.969083
C	6.695253	0.051469	0.703095
C	7.077427	0.919983	-0.345308
H	6.453936	2.404017	-1.778673
H	8.117435	0.935192	-0.662846
H	7.451018	-0.582499	1.162491
H	5.106380	-0.681481	1.973826

TS17

7	-2.301450	0.090129	-0.415816
6	-2.661547	1.178273	-0.189753
46	-3.108609	2.992387	0.206267
17	-3.612113	5.235923	0.790139
17	-5.165666	2.187753	1.101778
6	-2.462498	3.791826	-1.863703
7	-2.894796	4.478992	-2.711794
6	-3.474137	5.351356	-3.657579
6	-0.215162	-1.105335	-0.653656
6	0.394337	-2.487359	-0.852990
6	-1.741631	-1.209705	-0.614173
6	-0.070370	-3.461688	0.223021
6	-2.216084	-2.164936	0.481015
6	-1.592100	-3.540125	0.275618
1	-3.758471	6.282402	-3.157758
1	-4.363173	4.882852	-4.088994
1	-2.755423	5.569400	-4.452789
1	-2.109857	-1.561539	-1.589458
1	-3.311221	-2.217673	0.477206
1	-1.914301	-1.752851	1.454974
1	0.125055	-0.674009	0.300389
1	0.091484	-0.413678	-1.449351
1	0.105864	-2.870631	-1.844372
1	1.488323	-2.405679	-0.858101
1	-1.919921	-4.211198	1.079025
1	-1.969520	-3.973335	-0.663973
1	0.356599	-4.456885	0.044679
1	0.309278	-3.128812	1.201631
7	0.098138	2.945429	-0.289844
7	-0.761890	3.252270	0.709211
6	-0.073502	3.215044	1.839413

1	-0.246087	2.995533	-1.243438
1	-0.576342	3.419113	2.777438
6	1.360882	2.743045	0.176398
6	1.287324	2.902414	1.584675
6	2.553105	2.414042	-0.480430
6	2.446795	2.732325	2.361970
6	3.673791	2.250403	0.313365
6	3.624386	2.407333	1.716601
1	2.413051	2.852857	3.443064
1	4.535860	2.269420	2.294719
1	4.622120	1.993537	-0.155119
1	2.593786	2.292431	-1.560724

TS18

7	-1.858500	-1.518532	-0.240824
6	-2.374269	-0.386652	-0.379090
46	-2.622469	1.389539	-0.636045
17	-2.617373	3.686071	-1.064491
17	-4.923153	1.273692	-1.183959
6	-2.034404	-3.254608	1.492823
6	-2.752269	-4.538461	1.906100
6	-2.556295	-2.805055	0.136761
6	-4.264602	-4.355086	1.906787
6	-4.059065	-2.594229	0.118554
6	-4.760623	-3.878510	0.546712
1	-2.247365	-3.505850	-0.650176
1	-4.385044	-2.277383	-0.880423
1	-4.311360	-1.781519	0.819365
1	-2.234605	-2.460848	2.226933
1	-0.948018	-3.401915	1.455119
1	-2.479217	-5.347792	1.211998
1	-2.393026	-4.842042	2.896711
1	-5.843796	-3.709652	0.559212
1	-4.575740	-4.659007	-0.207284
1	-4.759804	-5.294057	2.183064
1	-4.541859	-3.617199	2.675276
7	0.193980	0.431249	-0.363438
7	-0.602771	1.512670	-0.147489
6	0.044952	2.548195	0.411337
1	-0.820799	-1.464600	-0.336534
1	-0.462396	3.479300	0.625244
6	1.420939	0.820359	0.067841
6	1.381000	2.156001	0.574867
6	2.628610	0.089856	0.067521
6	2.548791	2.765634	1.077876
6	3.753553	0.708477	0.569765
6	3.717329	2.035311	1.071285
1	2.524895	3.784949	1.460275
1	4.633898	2.479312	1.455941
1	4.699857	0.169225	0.580500
1	2.665999	-0.926764	-0.321623

TS19

7	-0.244653	-1.281194	-0.219043
6	0.395992	-0.151454	-0.067107
46	0.035281	1.759660	-0.359949
17	-0.018756	4.152454	-0.476686
17	-1.183085	1.538563	-2.394096
6	-1.587357	-2.146018	-2.093824
6	-3.005974	-2.307586	-2.627617
6	-1.607293	-1.417026	-0.755581
6	-3.902878	-3.016889	-1.619748
6	-2.485868	-2.130723	0.265936
6	-3.901322	-2.292294	-0.278585
1	-1.970373	-0.393656	-0.908765
1	-2.486778	-1.572783	1.212497
1	-2.057615	-3.124325	0.479958
1	-1.123815	-3.136928	-1.955985
1	-0.961842	-1.587566	-2.801174
1	-3.418204	-1.310319	-2.847492
1	-2.985114	-2.851054	-3.580566
1	-4.522092	-2.824205	0.453684
1	-4.352115	-1.295412	-0.405713
1	-4.926642	-3.095972	-2.007723
1	-3.544084	-4.048662	-1.474871
7	1.620621	-0.125507	0.566051
7	1.966732	1.192300	0.845562
6	3.129824	1.164897	1.442626
1	3.593358	2.089776	1.764813
6	2.614303	-0.998423	0.995224
6	3.607373	-0.175291	1.573776
6	2.763409	-2.382366	0.906125
6	4.769806	-0.745047	2.105245
6	3.926731	-2.922230	1.438405
6	4.914523	-2.120901	2.034841
1	5.537000	-0.117284	2.552711
1	5.808386	-2.591710	2.437104
1	4.076752	-3.998343	1.384349
1	2.030706	-3.026546	0.427935
1	0.157535	-2.141910	0.151253
6	-1.443877	1.682380	1.597632
7	-2.618768	1.717787	1.518502
6	-4.013074	1.783429	1.311704
1	-4.500349	0.921117	1.777245
1	-4.219462	1.784208	0.235566
1	-4.409401	2.702162	1.753729

TS20

6	4.381750	2.348850	-2.314032
6	3.301998	2.966432	-1.720151
6	2.105032	2.233142	-1.607566
6	2.044789	0.903351	-2.105553
6	3.138612	0.278034	-2.717555

6	4.297183	1.023193	-2.805169
6	0.841049	2.471366	-1.032204
7	0.094415	1.385817	-1.177688
7	0.796242	0.440376	-1.839167
7	-2.258846	0.895263	-0.063451
7	-2.504769	-0.380232	0.320121
6	-3.694672	-0.507616	0.910054
6	-4.304536	0.761638	0.932567
6	-3.352968	1.614794	0.298534
6	-5.523403	1.288259	1.401914
6	-5.761459	2.636829	1.230435
6	-4.811624	3.477044	0.599091
6	-3.609369	2.988156	0.128707
46	-1.101984	-1.789150	-0.133645
17	0.665748	-3.239233	-0.806132
6	0.192972	-1.013789	1.072355
7	1.041205	-0.580118	1.750945
6	2.209950	-0.136202	2.445214
6	3.116013	-1.330541	2.748508
6	4.392546	-0.855256	3.432872
6	5.119213	0.193134	2.598388
6	4.207637	1.373615	2.286014
6	2.925508	0.919745	1.600285
17	-2.644778	-2.773627	-1.634296
1	1.866709	0.315994	3.387004
1	2.249463	1.764932	1.419845
1	3.156923	0.474214	0.619768
1	3.356820	-1.830169	1.798468
1	2.575786	-2.057148	3.367105
1	4.140352	-0.428734	4.416512
1	5.042417	-1.717334	3.627245
1	4.720268	2.105203	1.647781
1	3.953777	1.898492	3.220406
1	6.021027	0.536654	3.120825
1	5.458373	-0.263510	1.655373
1	-4.039663	-1.474599	1.254438
1	-6.258812	0.645974	1.884283
1	-6.696181	3.068448	1.583967
1	-5.040930	4.535233	0.481360
1	-2.883881	3.637477	-0.360277
1	-0.935589	1.182870	-0.760589
1	0.437099	3.339741	-0.526858
1	3.367349	3.984378	-1.342453
1	5.322843	2.885223	-2.412024
1	5.176540	0.577373	-3.265305
1	3.074954	-0.740281	-3.091847
1	0.398839	-0.490068	-1.959122

TS21

7	-2.852589	-1.838985	-0.163104
6	-3.425784	-0.776350	-0.080002

46	-4.857158	0.533014	-0.020519
17	-6.456260	2.327666	0.056528
17	-6.508916	-1.158901	-0.252788
6	-2.523040	-4.020917	-1.214382
6	-3.080576	-5.432661	-1.357732
6	-3.429857	-3.176838	-0.328303
6	-3.286500	-6.088662	0.002813
6	-3.613079	-3.817403	1.044508
6	-4.170568	-5.229028	0.899721
1	-4.418235	-3.072189	-0.801566
1	-4.277144	-3.191990	1.655057
1	-2.637008	-3.845377	1.553831
1	-1.520944	-4.054380	-0.758562
1	-2.409497	-3.540831	-2.194923
1	-4.044786	-5.388462	-1.888726
1	-2.411031	-6.037468	-1.983705
1	-4.282247	-5.688826	1.890487
1	-5.182934	-5.171750	0.469062
1	-3.721700	-7.090141	-0.116653
1	-2.307368	-6.230485	0.488620
7	-2.172755	0.796090	0.155161
7	-3.205685	1.695977	0.166445
6	-2.760829	2.949681	0.216895
1	-3.450754	3.784685	0.234901
6	-1.021094	1.515260	0.193226
6	-1.350983	2.907400	0.240599
6	0.318579	1.084946	0.197734
6	-0.322577	3.867338	0.300304
6	1.302292	2.052103	0.251062
6	0.987536	3.431718	0.303357
1	-0.559408	4.929923	0.341236
1	1.797830	4.158079	0.346033
1	2.348770	1.749656	0.253989
1	0.560751	0.024288	0.159115
